# Lamin mutation location predicts cardiac phenotype severity: combined analysis of the published literature

**DOI:** 10.1136/openhrt-2018-000915

**Published:** 2018-10-25

**Authors:** Gabriella Captur, Eloisa Arbustini, Petros Syrris, Dina Radenkovic, Ben O'Brien, William J Mckenna, James C Moon

**Affiliations:** 1 UCL MRC Unit for Lifelong Health and Ageing, London, UK; 2 Barts Heart Centre, The Cardiovascular Magnetic Resonance Imaging Unit, St Bartholomew’s Hospital, London, UK; 3 Institute of Cardiovascular Science, University College London, London, UK; 4 Center for Inherited Cardiovascular Diseases, Foundation IRCCS Policlinico San Matteo, University of Pavia, Pavia, Italy; 5 Department of Perioperative Medicine, St Bartholomew's Hospital & Barts Heart Centre, London, UK; 6 William Harvey Research Institute, Charterhouse Square, Barts and the London School of Medicine and Dentistry, Queen Mary University of London, London, UK

**Keywords:** heart failure, systolic dysfunction, gene association

## Abstract

**Objective:**

Two *LMNA* genotype–phenotype cardiac correlations are reported: first, that cardiac involvement in multisystem laminopathies prevails with mutations upstream of the nuclear localisation signal (NLS); second, that worse outcomes occur with non-missense (compared with missense) mutations. We tested whether *LMNA* mutation DNA location and mutation subtype can predict phenotype severity in patients with lamin heart disease.

**Methods:**

We used a semantic workflow platform and manual electronic literature search to identify published *LMNA* mutations with cardiac-predominant phenotype. Hierarchical cluster analysis (HCA) assembled lamin heart disease into classes based on phenotype severity. 176 reported causative mutations were classified and any relationships to mutation location/subtype assessed by contingency analysis.

**Results:**

More adverse phenotype was associated with mutation location upstream of the NLS (p=0.014, OR 2.38, 95% CI 1.19 to 4.80) but not with non-missense mutations (p=0.337, OR 1.36, 95% CI 0.72 to 2.57), although an association with non-missense mutations was identified in a subcluster with malignant ventricular arrhythmia (p=0.005, OR 2.64, 95% CI 0.76 to 9.21). HCA limited to the 65 mutations described on ClinVar as pathogenic/likely pathogenic showed similar findings (upstream of NLS, p=0.030, OR 4.78, 95% CI 1.28 to 17.83; non-missense, p=0.121, OR 2.64, 95% CI 0.76 to 9.21) as did analysis limited to pathogenic/likely pathogenic variants according to the American College of Medical Genetics and Genomics standards.

**Conclusion:**

Cardiac patients with an *LMNA* mutation located upstream versus downstream of the NLS have a more adverse cardiac phenotype, and some missense mutations can be as harmful as non-missense ones.

**Key questions**

**What is already known about this subject?**

- Two *LMNA* genotype–phenotype cardiac correlations are currently reported: that cardiac involvement in multisystem laminopathies prevails with mutations upstream of the nuclear localisation signal (NLS), and that worse outcomes occur with non-missense (compared with missense) mutations.

**What does this study add?**

- More adverse phenotype was associated with mutation location upstream of the NLS but not with non-missense mutations, although an association with non-missense mutations was identified in a subcluster with malignant ventricular arrhythmia.

**How might this impact on clinical practice?**

- Cardiac patients with an *LMNA* mutation located upstream versus downstream of the NLS may have a more adverse cardiac phenotype, and some missense mutations can be as harmful as non-missense ones.

## Introduction

Lamins A and C are nuclear envelope proteins encoded by the *LMNA* gene (1q22). They are implicated in DNA replication, cell cycle regulation, chromatin organisation, differentiation maintenance, nuclear stability, pore positioning, gene expression and signal transduction. *LMNA* mutations cause laminopathies, a spectrum of multisystem diseases, including some types of muscular dystrophy, lipodystrophy and acrogeria syndromes.^1^ They also cause lamin heart disease,^2^ which accounts for up to 10% of dilated cardiomyopathies (DCM).^3^ Although there is significant pleiotropy of phenotypic expression in lamin heart disease,^2 4^ broadly it causes a malignant type of DCM with heart failure characterised by ventricular arrhythmias (VAs), cardiac conduction system disease (CCD) and an untreated sudden cardiac death (SCD) rate as high as 46%.^5^

**Figure 1:**
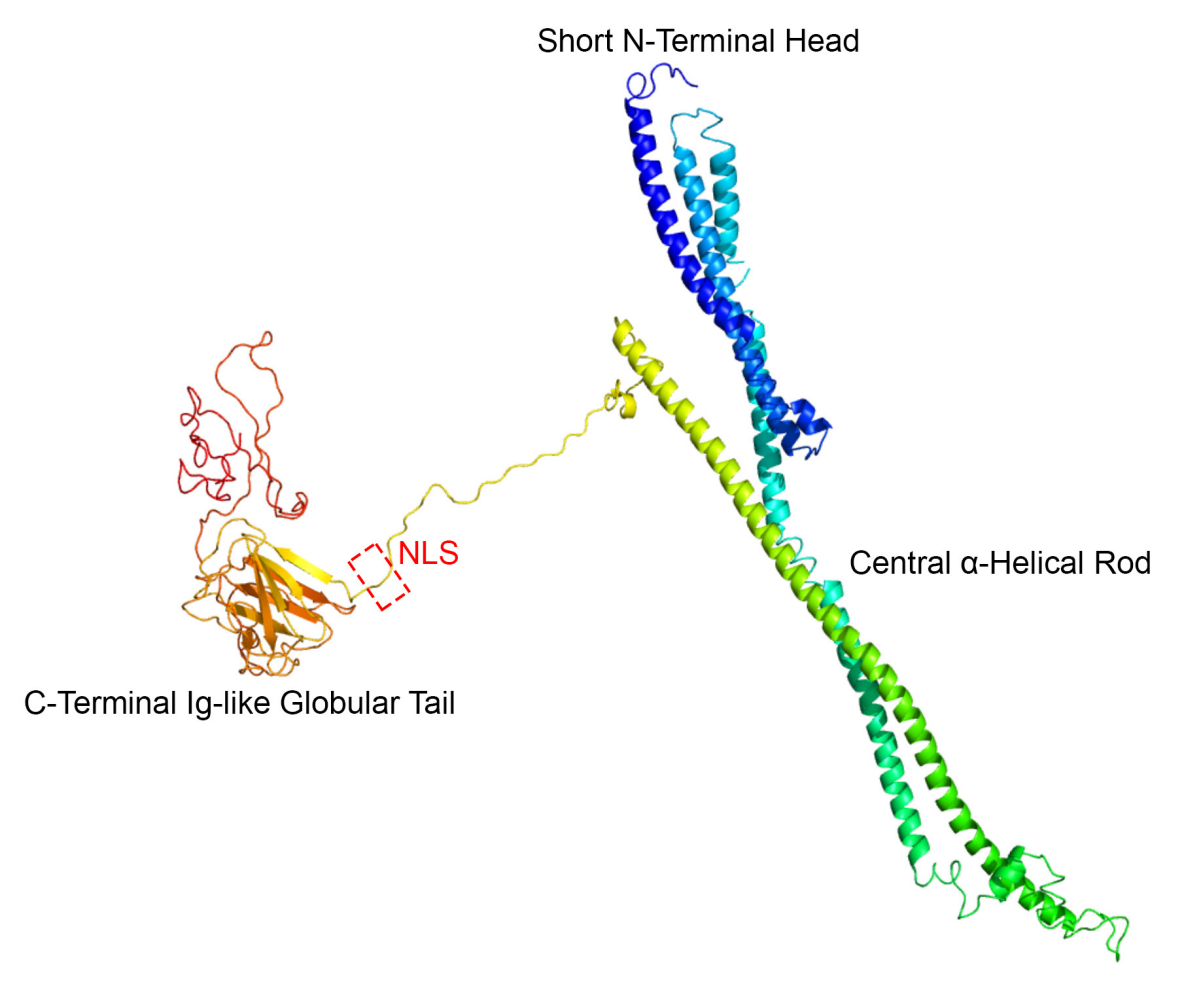
Three-dimensional model of wild-type lamin A. The *LMNA* gene uses an alternative 5’ splice site in intron 10 to generate four type A proteins: lamin A (major isoform, represented here, a.a. 664 protein with a molecular weight of 70 kDa), AΔ10 (minor isoform, missing exon 10), C (known as C1, a.a. 572) and C2 (only in germ cells). Each lamin protein has a tripartite domain organisation: short globular head, central α-helical rod and large immunoglobulin-like globular tail. Exons 7–9 code for tail domain sequences common to both lamins A and C. Highlighted in the model (red discontinuous box) are the five residues comprising the NLS sequence in exon 7 (a.a. 417–422) explaining its relationship to the other domains. The theoretical three-dimensional model was assembled using lamin A major isoform (UniProtKB—P02545) primary amino acid sequence data in FASTA format submitted to the Protein Homology/analogY Recognition Engine V.2.0 workspace^26^ (Phyre^2^) as an intensive job type. a.a., amino acid; NLS, nuclear localisation signal.

Being intermediate filament proteins, lamins A and C have three domains: a short globular N-terminal head, a central rod and a long globular C-terminal tail (figure 1). Between the rod and the tail lies a critical sequence—the nuclear localisation signal (NLS)—responsible for nuclear residency.^6^ Previous work has suggested that *LMNA* mutation position relative to the NLS predicts organ system involvement, with cardiac involvement prevailing with upstream mutations (ie, towards the N-terminal side).^7 8^ Three additional studies^9–11^ suggested worse outcomes in cardiac patients with non-missense as opposed to missense mutations. The latter genotype–phenotype relationship led the current European guidelines to treat non-missense mutations as one of four risk factors for SCD in a patient with DCM with an *LMNA* mutation (class IIa, level of evidence B^12^ for primary prevention with an implantable cardioverter defibrillator). The four risk factors are non-sustained ventricular tachycardia, left ventricular ejection fraction <45%, male gender and non-missense mutations.

Since these studies, many more mutations have been reported in the literature. Accordingly, we sought to systematically study whether *LMNA* mutation DNA location and subtype predict cardiac phenotype severity in patients with lamin heart disease.

## Methods

### Data collection

An up-to-date list of *LMNA* mutations reported to cause cardiac-predominant disease (online supplementary table 1) was assembled from a semantic search using a workflow developed inhouse that implemented a Simple Object Access Protocol-based service to retrieve publications in Europe PubMed Central via the Taverna Workbench (V.2.5.0).^13^ This system preserves the exact input, key intermediate, final output and relevant external data of the search, and securely captures the code, computational configurations, parameters and their provenance. The Taverna search was combined with a manual literature search of PubMed, MEDLINE (Ovid) and EMBASE (Ovid) for publications on *LMNA*-related DCM/SCD spanning 1999 until February 2018 (details in online supplementary methods). Additionally, we searched the National Center for Biotechnology Information Reference Sequences database^14^ (last accessed 30 January 2018). These data were manually cross-checked with the Universal Mutation Database-*LMNA* mutation database at www.umd.be/LMNA.

10.1136/openhrt-2018-000915.supp1 Supplementary data

The nomenclature found in online supplementary table 1 is provided according to the Human Genome Variation Society using the reference sequence *LMNA* lamin A (NM_170707.3), except in cases indicated, for which the original publication used the reference sequence for the lamin C transcript (NM_005572.3). Assertions of clinical significance per mutation were sought from the ClinVar archive^15^ and are reproduced in online supplementary table 1. Additionally, sequence variants were classified according to the American College of Medical Genetics and Genomics (ACMG) standards (www.acmg.net),^16 17^ and compared with entries in international databases, that is, The Human Gene Mutation Database^18^ (http://www.hgmd.cf.ac.uk). Each reported mutation was examined by the following in silico predictive algorithms: MutationTaster, PolyPhen-2, SIFT, PROVEAN and Condel (see online supplementary methods).

Mutation subtype was classified as non-missense (ins-del/truncating or mutations affecting splicing) or missense (single nucleotide substitutions) as previously described^10^ and as alluded to in the European guideline. We also separately explored a refined classification of mutation subtype based on predicted molecular consequences, thus aggregating missense | inframe insertion/deletion (termed missense/inframe ins/del) into one group and frameshift | nonsense | complex deletion | abnormal splicing mutations into another group, expected to have more adverse molecular consequences.

Mutation location was classified as upstream or downstream of the NLS with amino acid (a.a.) 417 as cut-off. For mutation location we also separately considered whether they occurred in the tail domain or upstream of the tail (a.a. 389 as cut-off, figure 1).

Phenotypic information per mutation was collected from the published literature to permit a MOGE(S) nosology classification.^19^ The MOGE(S) assigned to each mutation in online supplementary table 1 aggregates all the phenotypic traits of interest in index cases as well as family members bearing that particular mutation, from within the published literature. MOGE(S) is a five-letter phenotype–genotype standardised scheme for classifying cardiomyopathy endorsed by the World Heart Federation, similar to the tumour, node, metastases classification used in oncology. Its advantage is its ability to describe asymptomatic carriage, early disease forms and overlapping phenotypes. We scored for supraventricular arrhythmia; CCD (any degree of atrioventricular block as described in the original MOGE(S) scheme^19^); high creatine phosphokinase; juvenile onset (defined as age of first presentation <25 years); multisystem involvement (defined as any additional skeletal muscle, nervous, lipid, endocrine system manifestations); VA (defined as any type of non-life-threatening VA including ventricular ectopy); malignant ventricular arrhythmia ((MVA), defined as the potentially life-threatening VA, SCD, resuscitation or appropriate defibrillator therapy); and advanced heart failure ((aHF), defined as heart transplantation, death from end-stage heart failure or New York Heart Association functional classes III/IV). Although our study focused primarily on *LMNA* gene mutation carriers expressing a cardiac-predominant phenotype, some reported cases did secondarily have multisystem features, which is why we provisioned to score for multisystem involvement within the MOGE(S) as outlined above. Each subphenotypic attribute was scored as present (1)/absent (0), except for MVA and aHF which were scored as present (2)/absent (0) in proportion with their greater clinical importance. We provisioned for 0–0 match to be a ground of similarity across all categories.

To explore the prevalence of identified *LMNA* gene mutations in the general population, we mined data on the Genome Aggregation Database (gnomAD, accessed 19 December 2017, see online supplementary material 2), which includes 123 136 exome sequences and 15 496 whole-genome sequences from unrelated individuals^20^ (http://gnomad.broadinstitute.org/gene/ENSG00000160789). For each variant identified the allele frequency was recorded.

10.1136/openhrt-2018-000915.supp2 Supplementary data

### Statistical analyses

Unsupervised hierarchical cluster analysis (HCA) of the *LMNA* mutations with published and available phenotypic data was performed in R^21^ (packages ‘ggdendro’, ‘dendextend’). HCA segregated mutation carriers with similar multidimensional phenotypes into related regions of a dendrogram using a Euclidian distance metric and Ward’s minimum variance method that minimises the total within-cluster variance. At each step the pair of clusters with minimum between-cluster distance was merged. To demonstrate that identified clusters did not arise by chance (clustering noise or sampling error), we assessed the cophenetic correlation between each clustering result, using a total of seven possible cluster algorithms. We compared clustering solutions when cut to k=4 clusters, using the Fowlkes-Mallows Index (giving a value of 1 when the two clusters conformed, and 0 when they did not). We used the package ‘pvclust’ to compute approximately unbiased p values for all clusters contained in the clustering of original data by multiscale bootstrap resampling.

We used a 2×2 contingency analysis (χ^2^ or Fisher’s exact test as appropriate) to assess the relationship between HCA phenotype severity class, and (1) *LMNA* mutation subtype (missense vs non-missense; and missense/inframe ins/del vs others) and (2) *LMNA* mutation location (relative to the NLS and relative to the tail).

## Results

### Reported *LMNA* mutations

**Figure 2:**
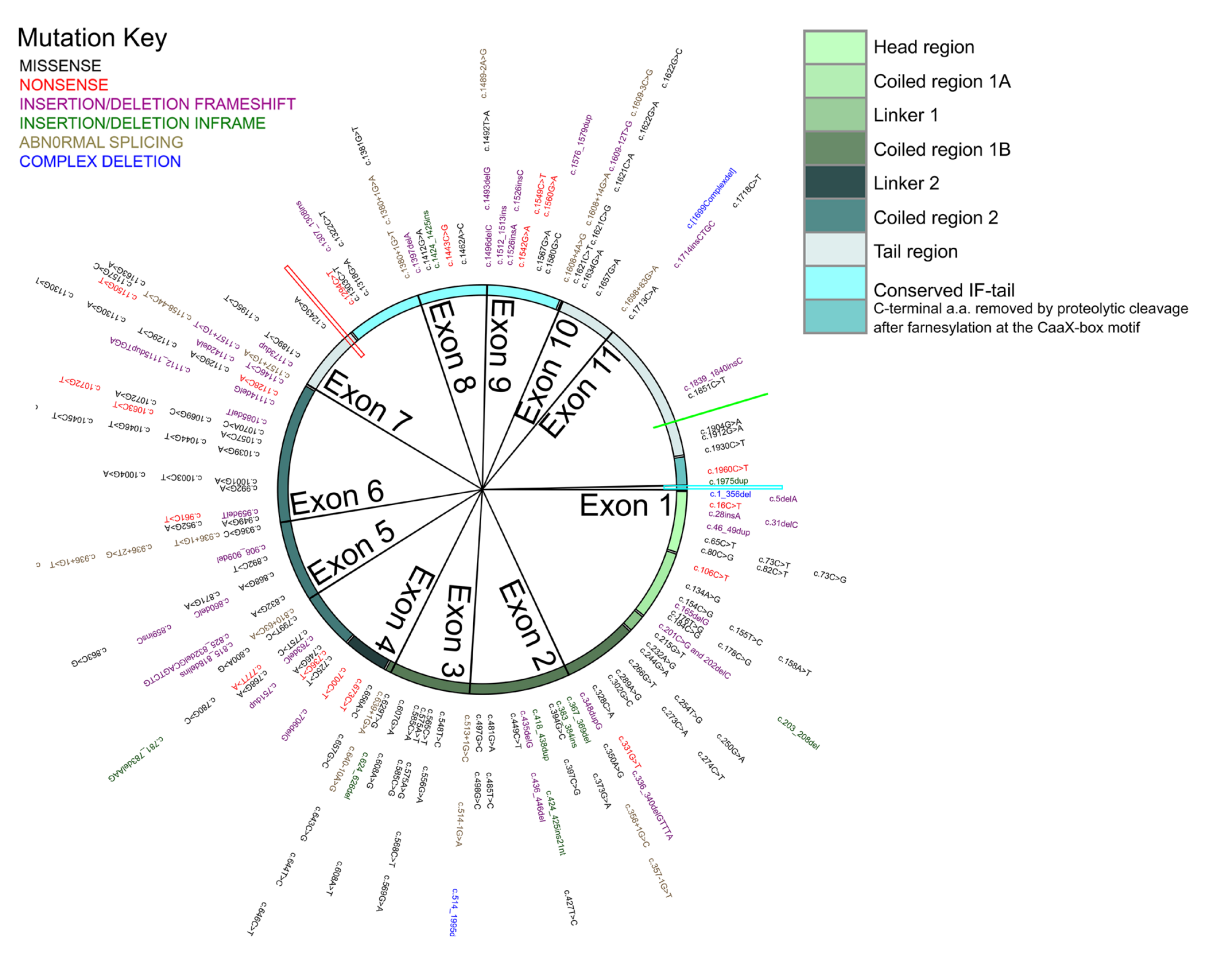
Mutational landscape in lamin heart disease. A Circos of mutation subtype in cDNA order along the *LMNA* gene. Each mutation is described in online supplementary table 1. Exon boundaries are demarcated by sector lines (exon 12 only holds one mutation and the sector is not resolvable). From outside to in: gene mutation variant (text colours indicate mutation type; key, top left), then *LMNA* cDNA location track. The outermost track is colour-coded to match structurally and functionally important domains of the lamin protein (key, top right). The position of the NLS (red spoke) relative to the conserved IF-tail is also indicated. To date no cardiac disease-causing *LMNA* mutation has been reported affecting the NLS (red spoke), the Ser 625 potential phosphorylation site (bright green spoke) or the lamin A specific –CaaX motif (a.a. 661–664, bright blue spoke). Circos was constructed in R (package ‘Circlize’). IF, intermediate filament. Other abbreviations as in figure 1.

A total of 199 cardiac-predominant *LMNA* mutations were identified, of which 109 (54.8%) were missense. Considering the refined classification, 111 mutations were in the missense/inframe ins/del group (63%). One hundred and seventy-six mutations (88%; figure 2) included sufficient published phenotypic information for a MOGE(S) classification to be used in the subsequent HCA. Of these, 65 carried a pathogenic/likely pathogenic assertion of clinical significance in ClinVar, while the remainder had conflicting interpretation of pathogencity or were of uncertain significance. Fifty-two (26%) published mutations were absent from ClinVar, and 45 (23%) although listed on ClinVar had no assertion of clinical significance provided. Of the 176 mutations used in the HCA, 145 carried a pathogenic/likely pathogenic assertion of clinical significance according to the ACMG standards.

Possibly as a result of lethality, we found that no cardiac disease-causing *LMNA* mutation has been reported affecting the (1) NLS (a.a. 417–422); (2) Ser 625 potential phosphorylation site; or (3) the lamin A specific –CaaX motif (a.a. 661–664; see figure 2), while only one mutation has been detected in the unique lamin C C-terminal sequence (VSGSRR, a.a. 566_572, NM_005572.3). Collectively these make up 2.9% of the entire lamin cDNA sequence.

### Phenotypic clustering

**Figure 3:**
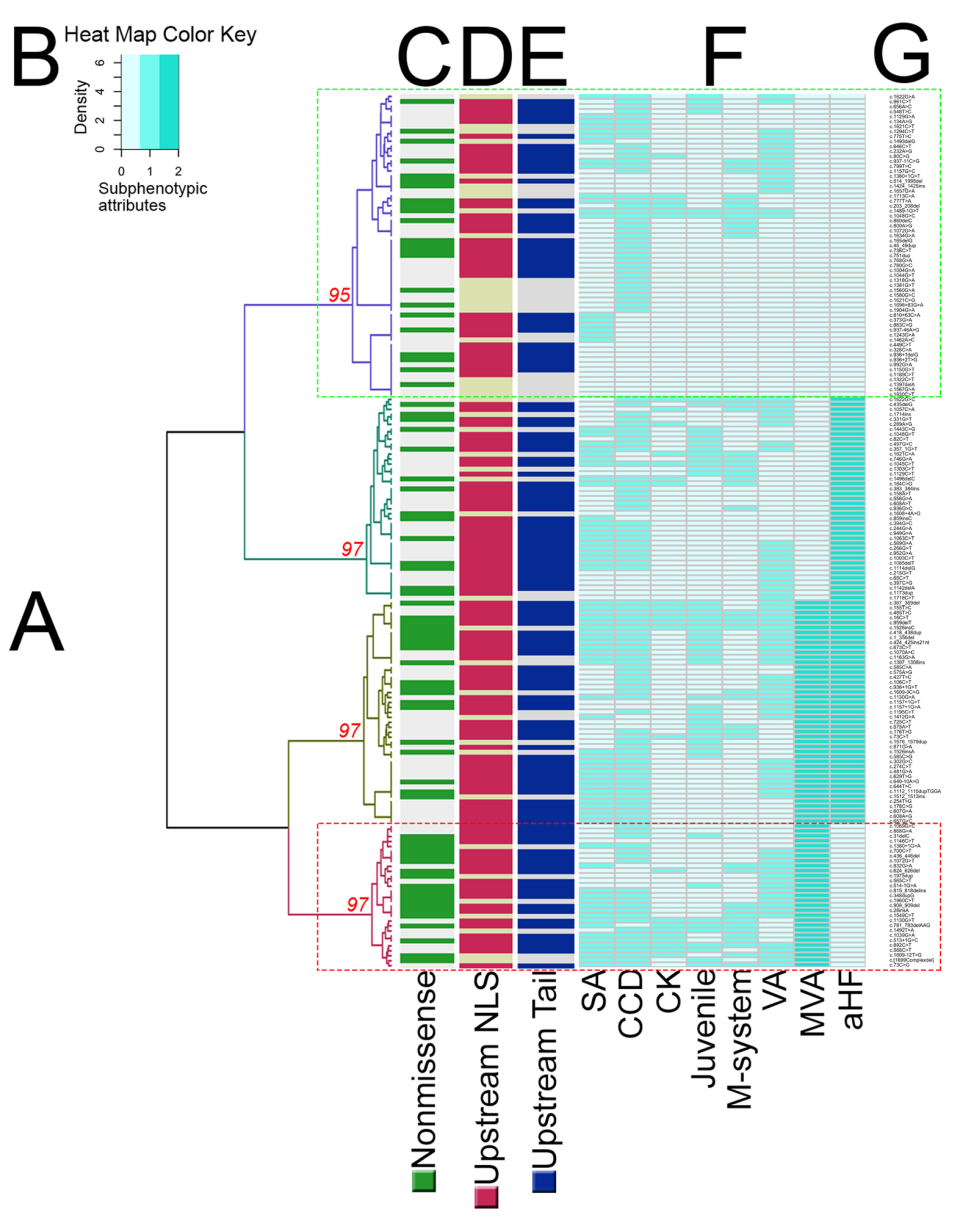
Hierarchical clustering of subphenotypic attributes in lamin heart disease. A dendrogram (A) was generated by unsupervised hierarchical clustering of 176 reported mutations. The horizontal limbs of the dendrogram represents the distance or dissimilarity between clusters. The vertical axis represents the objects and clusters. Fusion of two clusters is represented by a horizontal line splitting into two horizontal lines. The horizontal position of the split, shown by the short vertical line, summarises the dissimilarity between the two clusters. Looking at this dendrogram, one can see four clusters as four branches that occur at about the same horizontal distance. The phenotypic clusters were identified according to phenotype severity. The discontinuous green box (purple dendrogram at the top) highlights the mildest phenotype cluster, while the discontinuous red box (burgundy dendrogram at the bottom) highlights a cluster with MVA but not aHF that is enriched with non-missense mutations. AU p values per cluster are shown in red. Heat map key (B) denotes the relative intensities of each subphenotypic attribute scored in a sequential green colour gradient (extending over three units: 0, 1, 2). Values ≥95% are strongly supported by data. Coloured ribbons (C–E) summarise mutation-specific properties: (C) non-missense in green, missense in grey; (D) DNA location upstream of NLS in red, downstream in tan; (E) DNA location upstream of the tail in blue, downstream in tan. The subphenotype per mutation is presented in the form of a heatmap (F). The mutation list is provided on the far right (G). aHF, advanced heart failure defined as heart transplantation, death from end-stage heart failure or New York Heart Association functional class III/IV; AU, approximately unbiased; CCD, cardiac conduction system disease; CK, elevated creatine phosphokinase; juvenile, age of phenotypic penetrance <25 or ≥25 years, considering the earliest reported age of phenotypic expression in the proband or affected family members; M-system, multisystem involvement; MVA, malignant ventricular arrhythmia defined as sudden cardiac death, resuscitation or appropriate defibrillator therapy; SA, supraventricular arrhythmia; VA, any type of documented ventricular arrhythmia including ventricular ectopy. Other abbreviations as in figure 1.

**Figure 4:**
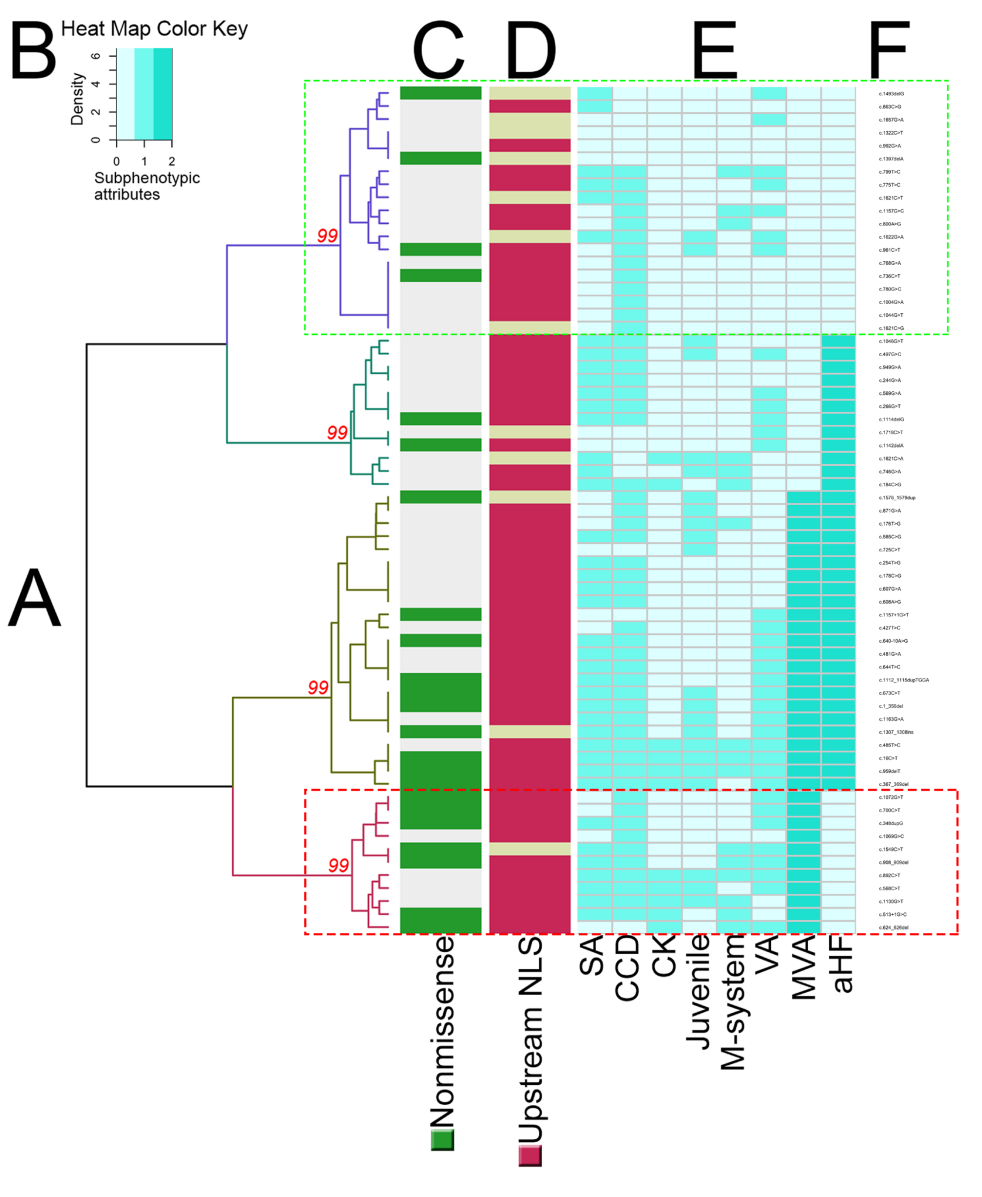
Hierarchical clustering of subphenotypic attributes in lamin heart disease limited to the pathogenic/likely pathogenic variants listed in ClinVar (n=65). Four significant phenotypic clusters (A) were identified according to phenotype severity. The discontinuous green box (purple dendrogram at the top) highlights the mildest phenotype cluster, while the discontinuous red box (burgundy dendrogram at the bottom) highlights a cluster with MVA but not aHF that is enriched with non-missense mutations. AU p values are shown in red. Heat map key (B) denotes the relative intensities of each subphenotypic attribute scored in a green sequential colour gradient (extending over three units: 0, 1, 2). Coloured ribbons (C, D) summarise mutation-specific properties: (C) non-missense in green, missense in grey; (D) DNA location upstream of NLS in red, downstream in tan. The subphenotype per mutation is presented in the form of a heatmap (E). The mutation list is provided on the far right (F). Abbreviations as in figure 1 and 3.

Unsupervised HCA consistently identified two main phenotypic groups (figures 3 and 4, and online supplementary table 2): 61 and 115 mutations in the clusters with less and more adverse phenotypes, respectively. Computational analysis suggested that the different clustering algorithms gave similar segregation results even when cut using the Fowlkes-Mallows Index (online supplementary table 3). HCA also consistently identified a smaller phenotypic subcluster (29 mutations; figure 3) characterised by MVA in the absence of aHF.

### Association between genotype and phenotype

There was an association between more adverse cardiac phenotype and *LMNA* DNA location upstream of the NLS (p=0.014, OR 2.38, 95% CI 1.19 to 4.80). HCA repeated for mutations relative to the tail domain similarly showed an association of more adverse cardiac phenotype with mutations upstream of the tail (p=0.013, OR 2.34, 95% CI 1.18 to 4.60) compared with those in the tail.

There was no association between non-missense mutation and more adverse cardiac phenotype (p=0.337, OR 1.36, 95% CI 0.72 to 2.57) except in the MVA subcluster, where non-missense mutations prevailed (p=0.005, OR 2.64, 95% CI 0.76 to 9.21). Repeating the HCA limited to the 65 *LMNA* mutations with pathogenic/likely pathogenic assertions listed in ClinVar showed a similar association between more adverse cardiac phenotype and mutation location upstream of the NLS (p=0.030, OR 4.78, 95% CI 1.28 to 17.83) and upstream of the tail (p=0.042, OR 3.89, 95% CI 1.10 to 13.81), but not with non-missense mutation subtype (p=0.121, OR 2.64, 95% CI 0.76 to 9.21), except again in the MVA subcluster (11 mutations), where non-missense mutations prevailed (p=0.043, OR 4.16, 95% CI 1.07 to 16.20). Repeating the HCA limited to the 145 *LMNA* mutations with pathogenic/likely pathogenic assertions according to the ACMG standards confirmed an association between more adverse cardiac phenotype and mutation location upstream of the NLS (p=0.012, OR 2.76, 95% CI 1.22 to 6.22) and upstream of the tail (p=0.028, OR 2.42, 95% CI 1.09 to 5.40), but not with non-missense mutation subtype (p=0.841, OR 1.08, 95% CI 0.52 to 2.24) except in the MVA subcluster (26 mutations), where non-missense mutations prevailed (p=0.032, OR 2.54, 95% CI 1.06 to 6.07). Repeating the HCA considering the 176 mutations reclassified into missense/inframe ins/del versus others showed no association between mutation subtype and cardiac phenotype severity (p=0.406, OR 1.32, 95% CI 0.69 to 2.53).

### Frequency of *LMNA* mutations in a general population database

Twenty-six of the 199 *LMNA* unique mutations presented in online supplementary table 1 (not counting the intronic variant, c.1158–44C>T—see below) were identified in gnomAD, amounting to a total of 704 *LMNA* mutations across the 138 632 gnomAD participants (0.51%). Eleven of these 26 mutations (42%) carried a pathogenic/likely pathogenic assertion on ClinVar. The majority (82%) of *LMNA* mutations identified in gnomAD were missense and located downstream of the NLS (83%). We did not count the intronic c.1158–44C>T mutation as it has only been described in one paper (online supplementary table 1), was identified in gnomAD at relatively high allele frequency (0.01069%) and is missing functional data.

## Discussion

Lamin heart disease accounts for 10% of DCM and is more malignant than typical DCM. By mining published mutations, and correlating mutation type and location with phenotype using a MOGE(S) scheme, we identified 199 published cardiac *LMNA* mutations. Some of these may be surprisingly common in the general population as we identified 704 in the 138 632 general population participants making up the gnomAD database.

Previously *LMNA* mutations upstream of the NLS (exons 1–6) were shown to have more cardiac organ involvement compared with other systems.^7^ Here we show for the first time in patients with lamin heart disease that such mutations have a more malignant cardiac phenotype compared with downstream mutations. Furthermore, it is not just mutations upstream of the NLS, but anywhere upstream of the tail (of the 176 unique mutations, 75% are upstream of the NLS; 72% are upstream of the tail). In support of this, *LMNA* mutations identified in the apparently healthy gnomAD population were rarely upstream of the NLS—eight times more likely to be located downstream (83 vs 621 allele counts). HCA data suggest that mutations upstream of the NLS, irrespective of mutation subtype, may be having a greater impact on protein structure and function relevant to the heart, and consequently on phenotypic expression, compared with mutations in and around the tail. One reason for this is that the region upstream of the NLS harbours the highly conserved rod domain, which is key to lamin protein assembly into mechanostructurally robust dimers (and then protofilaments, filament bundles, tangles and finally paracrystalline arrays). Another hypothesis is that while upstream mutations will invariably corrupt both isoforms, the lamin C isoform may be unscathed with more downstream mutations, thus potentially compensating for the mutant/deficient lamin A, mitigating phenotypic expression. The HCA data also show that while non-missense mutation subtype alone was insufficient to explain worse phenotype overall, it associated with MVA, as per the published literature and existing guideline. We go on to show that non-missense mutations are rare in the gnomAD population (12%) when compared with missense ones. Indeed, missense *LMNA* mutations distal to the NLS were the most common variants to be encountered in the gnomAD database search.

This work is the first time the MOGE(S) system was used to systematically classify mutation-specific morphofunctional subphenotypes across the published *LMNA* literature. We chose to employ hierarchical cluster agglomeration as this method is well suited for the interrogation of nominal data such as ours, by permitting a great many distance functions invented for nominal data, and theoretically more sound in this context than simply Euclidean distance. By combining a manual literature search with a transparent and reproducible^22^ search implemented through the Taverna workflow management system, we assembled a more up-to-date list of mutations that is sevenfold the number of mutations appraised by Pasotti *et* *al*,^9^ double that of van Rijsingen *et al* 10 and triple that of Kumar *et al* 11 (details provided in table 1). A significant number of *LMNA* mutations used in our analysis were not yet available to investigators listed in table 1 (112 variants were published after 2008; 19 variants were published after 2013), and even now more novel mutations continue to emerge.^23^

**Table 1:** Summary of studies exploring the relationship of non-missense *LMNA* mutations with outcomes in lamin heart disease

Publication	Study design	Sample size, NM|M	Total number of unique mutations studied, NM|M	Associated endpoint
Pasotti *et al* 9	Retrospective, one-centre	94, 35 | 59	23, 9 | 14	NM not associated with all events at UA. Splice site mutation associated with SCD at UA only.
van Rijsingen *et al* 2013* 27	Retrospective, eight-centre	269, 122 | 147	74, 36 | 38	NM not associated with MVA at UA. Considering date of birth until time of MVA (lifetime risk) NM shows independent association.
Kumar *et al* 28	Retrospective, five-centre	120, 51 | 69	55, 19 | 35 (+1 double M and NM)	NM independently associated with sustained VA and death.
Current HCA	Retrospective, /	/	176, 75 | 101	NM not associated with phenotype severity by HCA.

All studies regard exonic single nucleotide substitutions as missense mutations, and all others as non-missense.

*Mutation list for study participants was provided in a later separate publication (ref 27).

HCA, hierarchal cluster analysis; M, missense; MVA, malignant ventricular arrhythmia; NM, non-missense; SCD, sudden cardiac death; UA, univariate analysis; VA, ventricular arrhythmia.

The vast majority of disease-causing *LMNA* mutations are known to be sequence-level alterations and predominantly missense. Frameshift, nonsense, abnormal splicing and intragenic deletions and duplications, capable of resulting in a loss-of-function (haploinsufficiency), would be expected to have greater clinical impact when compared with single nucleotide substitutions. Data from three retrospective studies suggested this^9–11^ (table 1), but it is possible that the effect of some non-missense mutations may have been over-represented on account of inter-relatedness and clustering effects. Furthermore, some reported associations were tenuous or only apparent after reanalysis for lifetime risk of MVA.^10^ Non-missense mutations are currently considered a risk factor for SCD in the European guidelines,^12^ and indeed we confirmed an association between non-missense mutations and MVA in one subcluster. However, implicit in the guideline approach is the consequent treatment of all missense mutations as comparatively ‘less adverse’. While some such literature examples certainly exist, like the mild and late-onset phenotype reported for missense mutation p.Arg331Gln,^24^ other *LMNA* missense mutations have unequivocally been linked to highly adverse phenotypes (eg, p.Arg190Trp and p.Glu161Lys,^9^ both missense and located upstream of the NLS). Emerging data therefore suggest there may be a spectrum of phenotypic severity stemming from missense mutations of the *LMNA* gene with different mechanisms. A number of missense mutations are probably acting through a dominant negative pathway with adverse clinical consequences. For example in the case of the p.Ser143Pro missense mutation, the full length but aberrant lamin protein has been shown to accumulate, forming abnormal, round nucleoplasmic aggregates^25^ that would fit with a dominant negative effect. In keeping with this biological complexity, our HCA data suggest that the broad-based categorisation of mutations into ‘non-missense’ and ‘missense’ may be oversimplistic—yet as current guidelines suggest, there is a subset of patients with non-missense mutations at high risk of MVA; however, there are also a number of patients with missense mutations in the C-terminal exons of the *LMNA* gene expressing highly adverse phenotypes, which current guidelines may potentially misclassify if the four-variable SCD risk algorithm is applied, with consequent deferral of life-saving defibrillator implantation.

Limitations of our work include the reliance on reported mutations and clinical details from the literature, and possibly therefore inconsistent and incomplete data sets from different centres. While the genetic data published may have been precise, the scope and quality of the phenotype data and clinical investigations may not have been rigorous and occasionally incomplete, explaining our inability to include 12% of the 199 originally identified. Here we have focused on *LMNA* mutations with cardiac-predominant phenotype, as opposed to extending HCA to the much larger and broader multisystem laminopathies. We used an overall cardiac severity score rather than focusing on one specific aspect such as SCD as some published cohort studies may have done. Through the ACMG classification of variants, we incorporate into the HCA at least some modelling of predicted structural disruption by specific mutations on the tertiary structure of lamin A/C; however, the downstream effects on interacting molecules have not been modelled. Although the more malignant phenotypes (MVA and aHF) are known to be age-dependent, their individual age dependency was not scrutinised here on account of the heterogeneity of reported data in the published literature. Juvenile onset was therefore systematically captured instead. Future work should seek to rigorously investigate the relationship between the more adverse cardiac phenotype and gender.

## Conclusion

Lamin mutations upstream of the NLS or C-terminal tail domain associate with an overall more severe cardiac phenotype than those downstream, and some missense mutations can be as harmful as non-missense ones. Replication of findings in a larger prospective lamin heart disease cohort is needed to understand whether future cardiac guidelines should consider incorporating some of the more adverse missense mutations and mutation location (upstream of the NLS or C-terminal tail domain) for patient risk stratification.
