# Supplementary material for: Lamin mutation location predicts cardiac phenotype severity: combined analysis of the published literature
Source: Open Heart. 2018 Oct 25;5(2):e000915. doi: 10.1136/openhrt-2018-000915 (PMC6203068; doi:10.1136/openhrt-2018-000915)
Supplement: Supplementary data [file openhrt-2018-000915supp001.docx]

SUPPLEMENTARY MATERIAL FOR:

**Lamin mutation location predicts cardiac phenotype severity–combined analysis of the published literature**

Gabriella Captur MD PhD MRCP MSc,^1,2,3^ Eloisa Arbustini MD,^4^ Petros Syrris PhD,^3^ Dina Radenkovic BSc,^2^, Ben O’Brien MD PhD (Habil) FRCA FFICM MHBA,^5,6^ William J McKenna BA MD DSc FRCP FMedSci FESC FACC,^3^ James C Moon MD MBBS MRCP.^2,3^

1. UCL MRC Unit for Lifelong Health and Ageing, 33 Bedford Place, London WC1B 5JU, UK
2. Barts Heart Center, The Cardiovascular Magnetic Resonance Imaging Unit, St Bartholomew’s Hospital, West Smithfield, London, UK
3. Institute of Cardiovascular Science, University College London, Gower Street, London, WC1E 6BT, UK
4. Center for Inherited Cardiovascular Diseases, Foundation IRCCS Policlinico San Matteo, University of Pavia, Pavia, Italy
5. Department of Perioperative Medicine, St Bartholomew's Hospital & Barts Heart Centre, West Smithfield, London, EC1A 7BE, UK
6. William Harvey Research Institute, Charterhouse Square, Barts and the London School of Medicine and Dentistry, Queen Mary University of London, London, EC1M 6BQ, UK

**CORRESPONDENCE**

Prof James C Moon

Institute of Cardiovascular Science,

University College London,

Gower Street,

London WC1E 6BT, UK

E-mail: j.moon@ucl.ac.uk Phone No: +44 2034563081 Fax No: +0203 456 3086

**INDEX FOR SUPPLEMENTARY MATERIAL**

**Supplementary Methods.**

**Supplementary Table 1.** Genetic variants reported to date in association with lamin A/C heart disease, classified by MOGE(S) nosology.

**Supplementary Table 2.** Solutions from the various clustering algorithms.

**Supplementary Table 3.** Cluster conformity test results.

**References.**

**SUPPLEMENTARY METHODS**

**In silico predictive algorithms**

To permit classification of variants using the American College of Medical Genetics and Genomics (ACMG) standards each reported variant was examined by the following in silico predictive algorithms:

**MutationTaster**^1^ available at: http://www.mutationtaster.org/

**PolyPhen-2**^2^ available at: http://genetics.bwh.harvard.edu/pph2/

**SIFT**^3^ available at: http://sift.jcvi.org/

**PROVEAN**^3^ available at: http://provean.jcvi.org

**Condel**^4^ available at: http://bg.upf.edu/condel

**Manual search strategy**

First we identified a concept table of all free-text terms and controlled vocabulary terms pertinent to lamin heart disease organized as 4 concepts.

| **Concept 1** | **Concept 2** | **Concept 3** | **Concept 4** |
| --- | --- | --- | --- |
| #1 lamin | #1 dilated cardiomyopathy | #1 sudden cardiac death | #1 atrioventricular block |
| #2 laminopathy | #2 congestive dilated cardiomyopathy | #2 sudden cardiac arrest | #2 implantable cardioverter defibrillator |
| #3 LMNA | #3 hypokinetic non-dilated cardiomyopathy | #3 ventricular arrhythmia | #3 cardiac conduction system disease |
| #4 nuclear envelope | #4 heart failure | #4 malignant ventricular arrhythmia |  |
|  |  | #5 heart transplant |  |

In line with Cochrane guidance, we searched for each identified search term individually, then used the correct Boolean operators to combine the terms. This helped reduce human error and allowed us to see which search terms added value to the search and whether a particular search term produced too many irrelevant results. Here we provide one example of how we approached the search in PubMed:

**1.** Carried out separate searches for each free-text term (Text Words) and controlled vocabulary term (MeSH) in Concept 1 of Concept Table.

**2.** Combined all individual searches for Concept 1 with OR (used the Add link in the History to bring the individual searches back up into the Builder).

**3.** Repeated steps 1 and 2 for all other concepts.

**4.** Combined the OR searches for each concept with AND (used the Add link in the History to bring the individual searches back up into the Builder).

**SUPPLEMENTARY TABLE 1. Genetic variants reported to date in association with lamin A/C heart disease, classified by MOGE(S) nosology**

| **Variant (nucleotide change, predicted amino acid change)** | **Location: Variant type, molecular consequence*** | **Summative MOGE(S) classification**∞ | **ClinVar Clinical Significance** | **ACMG Assertion of Pathogenicity** | **Ref.#** |
| --- | --- | --- | --- | --- | --- |
| c.1_356del, p.Met1? | Exon 1: Large deletion (5’ region including exon 1), predicted protein truncation | M_D(SA, CCD, eHF, VA, MVA, <25)_ O_H(LV)_ G_AD_ E_G_ S_C-IV_ | Pathogenic | Pathogenic (Ia) | van Tintelen et al. 2007^5^ |
| c.5delA, p.Glu2GlyfsX94 | Exon 1: Single nucleotide insertion, frameshift and premature termination of translation | NA | N/A | Likely pathogenic (I) | van Rijsingen et al. 2013^6^ |
| c.16C>T, p.Gln6Ter | Exon 1: Single nucleotide substitution, nonsense | M_D(SA, CCD, eHF, VA, MVA, ↑CK, <25)_ O_H(LV)+M_ G_AD_ E_G_ S_C-IV_ | Pathogenic | Likely pathogenic (II) | Bécane et al. 2000^7^ |
| c.28insA, p.Thr10AsnfsX31 | Exon 1: Single nucleotide insertion, frameshift and premature termination of translation | M_D(SA, CCD, VA, MVA, <40)_ O_H(LV)+M_ G_AD_ E_G_ S_C-IV_ | Not provided | Likely pathogenic (I) | Sébillon et al. 2003^8^ |
| c.31delC, p.Arg11AlafsX85 (reported as p.Thr10fs85X) | Exon 1: Single nucleotide deletion, frameshift and premature termination of translation | M_D(SA, CCD, eHF, VA, MVA, ↑CK, <25, ≥25)_ O_H(LV)+M_ G_AD_ E_G_ S_C-NA_ | Not Provided | Pathogenic (Ia) | Pasotti et al. 2008^9^ |
| c.46_49dup, p.Ser18GlnfsX24 | Exon 1: Duplication (4 nucleotides), frameshift and premature termination of translation | M_D(CCD, MVA, <40)_ O_H(LV)_ G_AD_ E_G_ S_C-NA_ | N/A | Pathogenic (Ib) | Ho JCY et al. 2011^10^ |
| c.65C>T, p.Ser22Leu | Exon 1: Single nucleotide substitution, missense | M_D(eHF, VA, ≥25)_ O_H(LVRV)_ G_NA_ E_G_ S_C-IV_ | N/A | Likely pathogenic (II) | Pethig et al. 2004^11^ |
| c.73C>T, p.Arg25Cys | Exon 1: Single nucleotide substitution, missense | M_D(eHF, VA, ≥25)_ O_H(LV)_ G_AD_ E_G_ S_C-IV_ | Not provided | Likely pathogenic (II) | van Tintelen et al. 2007^12^~ |
| c.73C>G, p.Arg25Gly | Exon 1: Single nucleotide substitution, missense | M_D(CCD, eHF, MVA, ↑CK, <25)_ O_H(LV)+M_ G_AD_ E_G_ S_C-IV_ | Conflicting interpretations of pathogenicity: Pathogenic(1);Uncertain significance(1) | Likely pathogenic (II) | Yuan et al. 2009^13^ |
| c.80C>G, p.Thr27Ser | Exon 1: Single nucleotide substitution, missense | M_D(CCD, VA, ↑CK, ≥25)_ O_H(LVRV, A)_ G_AD_ E_G_ S_C-I_ | Uncertain significance | Likely pathogenic (II) | Parnham et al. 2015^14^ |
| c.82C>T, p.Arg28Trp | Exon 1: Single nucleotide substitution, missense | M_D(CCD, eHF, <40)_ O_H(LV)_ G_AD_ E_G_ S_C-IV_ | Not provided | Likely pathogenic (II) | Pasotti et al. 2008^9^ |
| c.106C>T, p.Gln36Ter | Exon 1: Single nucleotide substitution, nonsense | M_D(CCD, VA, MVA, ≥25)_ O_H(LV)_ G_AD_ E_G_ S_C-III_ | Not provided | Likely pathogenic (II) | Arbustini et al. 2009^15^ |
| c.134A>G, p.Tyr45Cys | Exon 1: Single nucleotide substitution, missense | M_D(SA, CCD)_ O_H(LV)+M_ G_AD_ E_G_ S_C-NA_ | Uncertain significance | Likely pathogenic (II) | Arola et al. 2004^16^ |
| c.154C>G, p.Leu52Val | Exon 1: Single nucleotide substitution, missense | NA | Likely pathogenic | Likely pathogenic (II) | https://www.ncbi.nlm.nih.gov/clinvar/variation/48042/ |
| c.155T>C , p.Leu52Pro | Exon 1: Single nucleotide substitution, missense | M_D(SA, CCD, VA, MVA, ↑CK, <25)_ O_H(LV)_ G_AD_ E_G_ S_C-III_ | Not provided | Likely pathogenic (II) | Rudenskaya et al. 2008^17^ |
| c.158A>T, p.Glu53Val | Exon 1: Single nucleotide substitution, missense | M_D(CCD, eHF, ≥25)_ O_H(LV)_ G_DN_ E_G_ S_C-IV_ | Not provided | Likely pathogenic (II) | Song et al. 2007^18^ |
| c.165delG, p.Asn56ThrfsX40 | Exon 1: Single nucleotide deletion, frameshift and premature termination of translation | M_D(CCD, ≥25)_ O_H(LV)_ G_AD_ E_G_ S_C-II_ | N/A | Likely pathogenic (I) | Verga et al. 2003^19^ |
| c.176T>G, p.Leu59Arg | Exon 1: Single nucleotide substitution, missense | M_D(MVA, <25)_ O_H(LV) + L + E_ G_AD_ E_G_ S_C-IV_ | Pathogenic | Likely pathogenic (II) | McPherson E et al. 2009^20^ |
| c.178C>G, p.Arg60Gly | Exon 1: Single nucleotide substitution, missense | M_D(SA, CCD, eHF, MVA, ↑CK, ≥25)_ O_H(LV)_ G_AD_ E_G_ S_C-IV_ | Pathohenic | Pathogenic (II) | Fatkin et al. 1999^21^ |
| c.184C>G, p.Arg62Gly | Exon 1: Single nucleotide substitution, missense | M_D(SA, CCD, ↑CK, ≥25)_ O_H(LV, A)+M+L+E_ G_AD_ E_G_ S_C-IV_ | Pathogenic: Familial partial lipodystrophy 2, not provided | Likely pathogenic (II) | Garg et al. 2002^22^ |
| c.201C>G and 202delC, p.Thr64SerfsX32 (reported as c.291C>G and 292delC) | Exon 1: Single nucleotide substitution and single nucleotide deletion, frameshift and premature termination of translation | NA | N/A | Likely pathogenic (I) | van Rijsingen et al. 2013^6^ |
| c.203_208delAGGTGG, p.Glu68_Val69del | Exon 1: Deletion (6 nucleotides), inframe deletion of 2 amino acids | M_E(CCD, ↑CK, ≥25)_ O_H(LV)+M_ G_AD_ E_G_ S_C-I_ | Not provided | Likely pathogenic (II) | Arbustini et al. 2007^23^ |
| c.215G>T, p.Arg72Leu | Exon 1: Single nucleotide substitution, missense | M_D(eHF, VA)_ O_H(LV)_ G_AD_ E_G_ S_C-IV_ | Uncertain significance | VUS - not enough evidence | Kumar et al. 2016^24^ |
| c.232A>G, p.Lys78Glu (reported as c.444A>G) | Exon 1: Single nucleotide substitution, missense | M_D(CCD, VA, ≥25)_ O_H(LV)_ G_AD_ E_G_ S_C-IV_ | Uncertain significance | VUS - not enough evidence | Kourgiannidis et al. 2013^25^ |
| c.244G>A, p.Glu82Lys | Exon 1: Single nucleotide substitution, missense | M_D(SA, CCD, eHF, ≥25)_ O_H(LV)_ G_AD_ E_G_ S_C-IV_ | Pathogenic | Likely pathogenic (III) | Wang et al. 2006^26^ |
| c.250G>A, p.Glu84Lys | Exon 1: Single nucleotide substitution, missense | NA | N/A | Likely pathogenic (II) | van Rijsingen et al. 2013^6^ |
| c.254T>G, p.Leu85Arg | Exon 1: Single nucleotide substitution, missense | M_D(SA, CCD, eHF, MVA, ≥25)_ O_H(LV)_ G_AD_ E_G_ S_C-IV_ | Pathogenic | Likely pathogenic (II) | Fatkin et al. 1999^21^ |
| c.266G>T, p.Arg89Leu | Exon 1: Single nucleotide substitution, missense | M_D(SA, CCD, eHF, VA)_ O_H(LV)_ G_AD_ E_G_ S_C-IV_ | Pathogenic | Likely pathogenic (II) | Taylor et al. 2003^27^ |
| c.273C>A, p.Thr91Thr | Exon 1: Single nucleotide substitution, missense | NA | N/A | VUS - not enough evidence | Fokkema et al. 2005^28^ |
| c.274C>T, p.Leu92Phe | Exon 1: Single nucleotide substitution, missense | M_D(SA, CCD, eHF, VA, MVA< ≥25)_ O_H(LV)_ G_AD_ E_G_ S_C-NA_ | Not provided | Likely pathogenic (II) | Millat et al. 2009^29^, Chami et al. 2012^30^ |
| c.289A>G, p.Lys97Glu | Exon 1: Single nucleotide substitution, missense | M_D(CCD, eHF, VA, ↑CK, ≥25)_ O_H(LV)_ G_AD_ E_G_ S_C-IV_ | Not provided | Likely pathogenic (II) | Arbustini et al. 2002^31^ |
| c.302G>C, p.Arg101Pro | Exon 1: Single nucleotide substitution, missense | M_D(SA, CCD, eHF, VA, MVA, ≥25)_ O_H(LV)_ G_AD_ E_G_ S_C-IV_ | Uncertain significance | Likely pathogenic (II) | Parks et al. 2009^32^ |
| c.328C>A, p.Arg110Ser | Exon 1: Single nucleotide substitution, missense | M_D_ O_H(LV)_ G_AD_ E_G_ S_C-NA_ | Uncertain significance | VUS - not enough evidence | Botto et al. 2011^33^ |
| c.331G>T, p.Glu111Ter | Exon 1: Single nucleotide substitution, nonsense | M_D(CCD, eHF, VA, ≥25)_ O_H(LV)_ G_AD_ E_G_ S_C-IV_ | N/A | Pathogenic (Ia) | Arbustini et al. 2002^31^ |
| c.336_340delGTTTA, p.Phe113GlyfsX12 | Exon 1: Deletion (5 nucleotides), frameshift and premature termination of translation | NA | N/A | Pathogenic (Ic) | van Rijsingen et al. 2013^6^ |
| c.348dupG, p.Lys117GlufsX10 | Exon 1: Single nucleotide duplication, frameshift and premature termination of translation | M_D(SA, CCD, VA, MVA, ≥25)_ O_H(LV)_ G_AD_ E_G_ S_C-NA_ | Pathogenic | Pathogenic (Ia) | Pan et al. 2009^34^ |
| c.350A>G, p.Lys117Arg | Exon 1: Single nucleotide substitution, missense | NA | Conflicting interpretations of pathogenicity: Likely pathogenic(1);Uncertain significance(1) | VUS - not enough evidence | van Rijsingen et al. 2013^6^ |
| c.356+1G>C | Intron 1: Single nucleotide substitution, predicted abnormal splicing | NA | Likely pathogenic | VUS - not enough evidence | van Rijsingen et al. 2013^6^ |
| c.357−1G>T | Intron 1: Single nucleotide substitution, predicted abnormal splicing | M_D(SA, CCD, eHF, VA, <25)_ O_H(LV)_ G_AD_ E_G_ S_C-IV_ | Not provided | VUS - not enough evidence | Parks et al. 2009^32^ |
| c.367–369delAAG, p.Lys123del | Exon 2: Deletion (3 nucleotides), inframe deletion of single amino acid | M_D(SA, CCD, VA, MVA, ↑CK, <25)_ O_H(LV)_ G_AD_ E_G_ S_C-IV_ | Likely pathogenic | Likely pathogenic (II) | Keller et al. 2012^35^ |
| c.373G>A, p.Gly125Ser | Exon 2: Single nucleotide substitution, missense | M_E(SA)_ O_H(LV)_ G_DN_ E_G_ S_C-NA_ | Not provided | VUS - not enough evidence | Brauch et al. 2010^36^ |
| c.383_384ins24bp, p.Ile128-Ala129insRVTLISSR | Exon 2: Insertion (24 nucleotides), inframe insertion of 8 amino acids | M_D(CCD)_ O_H(LV)_ G_AD_ E_G_ S_C-III_ | N/A | Pathogenic (Ic) | Millat et al. 2009^29^ |
| c.394G>C, p.Ala132Pro | Exon 2: Single nucleotide substitution, missense | M_D(SA, CCD, eHF, ≥25)_ O_H(LV)_ G_AD_ E_G_ S_C-IV_ | Not provided | Likely pathogenic (II) | Kärkkäinen et al. 2006^37^ |
| c.397C>G,p.Arg133Gly | Exon 2: Single nucleotide substitution, missense | M_D(eHF, VA)_ O_H(LV)_ G_NA_ E_G_ S_C-IV_ | N/A | VUS - not enough evidence | Kumar et al. 2016^24^ |
| c.418–438dup, p.Leu140_Ala146dup | Exon 2: Duplication (21 nucleotides), inframe duplication of 7 amino acids | M_D(SA, CCD, eHF, VA, MVA, <25)_ O_H(LVRV)+M_ G_AD_ E_G_ S_C-IV_ | N/A | Pathogenic (II) | Forleo et al. 2015^38^ |
| c.424_425ins21nt, p.Leu141_Asn142insLysAspLeuAspAlaLeuLeu | Exon 2: Duplication (21 nucleotides), inframe duplication of 7 amino acids | M_D(SA, CCD, eHF, VA, MVA, <25)_ O_H(LV)_ G_AD_ E_G_ S_C-IV_ | N/A | VUS - not enough evidence | Perrot et al. 2006^39^ |
| c.427T>C, p.Ser143Pro | Exon 2: Single nucleotide substitution, missense | M_D(CCD, eHF, VA, MVA)_ O_H_ G_NA_ E_G_ S_C-IV_ | Pathogenic | Pathogenic (II) | Kärkkäinen et al. 2004^40^ |
| c.435delG, p.Ala146ProfsX2 | Exon 2: Single nucleotide deletion, frameshift and premature termination of translation | M_D(CCD, eHF, VA, <25)_ O_H(LV)+M_ G_AD_ E_G_ S_C-IV_ | N/A | Pathogenic (Id) | Simms-Williams et al. 2013^41^ |
| c.436_446delGCCGCACTGAG, p.Ala146HisfsX5 | Exon 2: Deletion (11 nucleotides), frameshift and premature termination of translation | M_E(CCD, VA, MVA)_ O_H(LV)_ G_AD_ E_G_ S_C-I_ | N/A | Pathogenic (Ib) | Nishi et al. 2016^42^ |
| NM_005572.3:c.449C>T, p.Thr150Ile | Exon 2: Single nucleotide substitution, missense | M_D_ O_H(LV)_ G_NA_ E_G_ S_C-NA_ | N/A | VUS - not enough evidence | Hirlte-Lewis et al. 2013^43^ |
| c.459_462delTGAG, p.Ser153ArgfsX22 | Exon 2: Deletion (4 nucleotides), frameshift and premature termination of translation | NA | N/A | Pathogenic (Ic) | van Rijsingen et al. 2013^6^ |
| c.481G>A, p.Glu161Lys | Exon 2: Single nucleotide substitution, missense | M_D(SA, CCD, eHF, VA, MVA, ≥25)_ O_H(LV)_ G_AD_ E_G_ S_C-IV_ | Pathogenic | Pathogenic (II) | Sébillon et al. 2003^8^ |
| c.485T>C, p.Leu162Pro | Exon 2: Single nucleotide substitution, missense | M_D(SA, VA, MVA, CCD, eHF, ↑CK, <25)_ O_H(LV)+M_ G_AD_ E_G_ S_C-IV_ | Likely pathogenic | Likely pathogenic (II) | Brodsky GL et al. 2000^44^ |
| c.497G>C, p.Arg166Pro | Exon 2: Single nucleotide substitution, missense | M_D(SA, CCD, eHF, VA, <25)_ O_H(LV)_ G_AD_ E_G_ S_C-IV_ | Pathogenic/Likely pathogenic | Likely pathogenic (II) | Parks et al. 2009^32^ |
| NM_005572.3:c.513+1G>C | Intron 2: Single nucleotide substitution, predicted abnormal splicing | M_D(SA, MVA, CCD, ↑CK, ≥25)_ O_H(LV, RV, A)+M+N_ G_AD_ E_G_ S_C-II_ | Likely pathogenic | Likely pathogenic (II) | Chen C et al. 2013^45^ |
| c.514-1G>A | Intron 2: Single nucleotide substitution, predicted abnormal splicing | M_D(VA, MVA, <40)_ O_H(LV)_ G_DN_ E_G_ S_C-IV_ | Not provided | VUS - not enough evidence | Pasotti et al. 2008^9^ |
| c.514_1995del, p.Leu172fsX22 | Exon 3: Deletion from exon 3 to 12, frameshift and premature termination of translation | M_D(VA, ≥25)_ O_H(LV)_ G_DN_ E_G_ S_C-II_ | N/A | Pathogenic (Ic) | Gupta et al. 2010^46^ |
| c.548T>C, p.Leu183Pro | Exon 3: Single nucleotide substitution, missense | M_D(CCD, <40)_ O_H(LV)_ G_DN_ E_G_ S_C-NA_ | Not provided | Likely pathogenic (II) | Pasotti et al. 2008^9^ |
| c.556G>A, p.Glu186Lys | Exon 3: Single nucleotide substitution, missense | M_D(CCD, eHF, ≥25)_ O_H(LV)_ G_DN_ E_G_ S_C-IV_ | Not provided | Pathogenic (II) | Song et al. 2007^18^ |
| c.565C>T, p.Arg189Trp | Exon 3: Single nucleotide substitution, missense | M_D(VA, MVA, ≥25)_ O_H(LV)_ G_AD_ E_G_ S_C-II_ | Uncertain significance | Pathogenic (II) | Botto et al. 2010^47^ |
| c.[1699 to 183_1699–160inv24; 568_1699–184del; 1699 to 159_1995+6997del] | Exon 3 + 10: Complex double deletion with break point (24-bp inversion flanked by 3.8-kb deletion upstream & 7.8-kb deletion downstream) | M_D(SA, CCD, VA, MVA, <25)_ O_H(LV, A)_ G_AD_ E_G_ S_C-NA_ | N/A | Pathogenic (Ia) | Marsman et al., 2011^48^ |
| c.568C>T, p.Arg190Trp | Exon 3: Single nucleotide substitution, missense | M_D(SA, CCD, VA, MVA, ↑CK, <25)_ O_H(LV)_ G_AD_ E_G_ S_C-NA_ | Pathogenic | Pathogenic (IIIb) | Arbustini et al. 2002^31^ |
| c.569G>A, p.Arg190Gln | Exon 3: Single nucleotide substitution, missense | M_D(SA, CCD, eHF, VA, ≥25)_ O_H(LV)_ G_AD_ E_G_ S_C-IV_ | Pathogenic | Likely pathogenic (II) | Parks et al. 2009^32^ |
| c.575A>T, p.Asp192Val | Exon 3: Single nucleotide substitution, missense | M_D(eHF, MVA, <25)_ O_H(LV)_ G_AD_ E_G_ S_C-IV_ | Not provided | Pathogenic (II) | Subramanyam et al. 2010^49^ |
| c.575A>G, p.Asp192Gly | Exon 3: Single nucleotide substitution, missense | M_D(CCD, eHF, MVA)_ O_H(LV)_ G_AD_ E_G_ S_C-IV_ | Not provided | Pathogenic (II) | Sylvius et al. 2005^50^ |
| c.585C>A, p.Asn195Lys | Exon 3: Single nucleotide substitution, missense | M_D(CCD, eHF, MVA, ≥25)_ O_H(LV)_ G_AD_ E_G_ S_C-IV_ | Not provided | Likely pathogenic (II) | van Tintelen et al. 2007^12^ |
| c.585C>G, p.Asn195Lys | Exon 3: Single nucleotide substitution, missense | M_D(SA, CCD, eHF, MVA, <25)_ O_H(LV, A)_ G_AD_ E_G_ S_C-IV_ | Pathogenic | Pathogenic (II) | Fatkin et al. 1999^21^ |
| c.607G>A, p.Glu203Lys | Exon 3: Single nucleotide substitution, missense | M_D(SA, CCD, eHF, MVA)_ O_H(LV)_ G_AD_ E_G_ S_C-IV_ | Pathogenic | Pathogenic (II) | Jakobs et al. 2001^51^ |
| c.608A>G, p.Glu203Gly | Exon 3: Single nucleotide substitution, missense | M_D(SA, CCD, eHF, MVA, ≥25)_ O_H(LV)_ G_AD_ E_G_ S_C-IV_ | Pathogenic | Pathogenic (II) | Fatkin et al. 1999^21^ |
| c.608A>T, p.Glu203Val | Exon 3: Single nucleotide substitution, missense | M_D(CCD, eHF)_ O_H(LV)_ G_AD_ E_G_ S_C-IV_ | Not provided | Pathogenic (II) | Perrot et al. 2009^52^ |
| c.624_626delAAG, p.Lys208del | Exon 3: Deletion (3 nucleotides), inframe deletion of single amino acid | M_D(VA, MVA, ↑CK, ≥25)_ O_H(LV)+M_ G_AD_ E_G_ S_C-NA_ | Pathogenic | Pathogenic (IIIb) | van Tintelen et al. 2007^12^~ |
| c.629T>G, p.Ile210Ser | Exon 3: Single nucleotide substitution, missense | M_D(SA, CCD, eHF, VA, MVA, ≥25)_ O_H(LV)_ G_AD_ E_G_ S_C-IV_ | Uncertain significance | Likely pathogenic (II) | Parks et al. 2009^32^ |
| c.639+1G>A | Intron 3: Single nucleotide substitution, predicted abnormal splicing | NA | N/A | VUS - not enough evidence | van Rijsingen et al. 2013^6^ |
| c.640-10A>G | Intron 3: Single nucleotide substitution, predicted abnormal splicing | M_D(SA, CCD, eHF, VA, MVA, ≥25)_ O_H(LV)_ G_AD_ E_G_ S_C-IV_ | Pathogenic/Likely pathogenic | Likely pathogenic (II) | Otomo et al. 2005^53^ |
| c.643C>G, p.Leu215Val | Exon 4: Single nucleotide substitution, missense | NA | Uncertain significance | VUS - not enough evidence | van Rijsingen et al. 2013^6^ |
| c.644T>C, p.Leu215Pro | Exon 4: Single nucleotide substitution, missense | M_D(SA, CCD, eHF, VA, MVA, ≥25)_ O_H(LV)_ G_AD_ E_G_ S_C-IV_ | Pathogenic | Pathogenic (II) | Hershberger et al. 2002^54^ |
| c.646C>T, p.Arg216Cys | Exon 4: Single nucleotide substitution, missense | M_D(CCD, VA, ≥25)_ O_H(LV)_ G_AD_ E_G_ S_C-I_ | Conflicting interpretations of pathogenicity: Likely pathogenic(1);Uncertain significance(4) | VUS - not enough evidence | van Rijsingen et al. 2013^6^, Rasmussen et al. 2017^55^ |
| c.656A>C, p.Lys219Thr | Exon 4: Single nucleotide substitution, missense | M_D(CCD, <40)_ O_H(LV)_ G_AD_ E_G_ S_C-NA_ | Uncertain significance | Likely pathogenic (II) | Pasotti et al. 2008^9^ |
| c.657G>C, p.Lys219Asn | Exon 4: Single nucleotide substitution, missense | M_D(SA, CCD, eHF, MVA, ≥25)_ O_H(LV)_ G_AD_ E_G_ S_C-NA_ | N/A | Pathogenic (IIIb) | Arbustini et al. 2007^23^ |
| NM_005572.3(LMNA):c.673C>T, p.Arg225Ter | Exon 4: Single nucleotide substitution, nonsense | M_D(SA, CCD, eHF, VA, MVA, <25)_ O_H(LV)_ G_AD_ E_G_ S_C-IV_ | Pathogenic | Pathogenic (Ia) | Jakobs et al. 2001^51^ |
| c.700C>T, p.Gln234Ter | Exon 4: Single nucleotide substitution, nonsense | M_D(CCD, VA, MVA, ≥25)_ O_H(LV)_ G_AD_ E_G_ S_C-IV_ | Pathogenic/Likely pathogenic | Likely pathogenic (II) | Parks et al. 2009^32^ |
| c.706delG, p.Glu236SerfsX262 | Exon 4: Single nucleotide deletion, frameshift and premature termination of translation | NA | N/A | Pathogenic (Ic) | van Rijsingen et al. 2013^6^ |
| c.725C>T, p.Ala242Val | Exon 4: Single nucleotide substitution, missense | M_D(MVA, ≥25)_ O_H(LRV)_ G_AD_ E_G_ S_C-IV_ | Pathogenic/Likely pathogenic | Likely pathogenic (II) | https://www.ncbi.nlm.nih.gov/clinvar/variation/48076 |
| c.736C>T, p.Gln246Ter | Exon 4: Single nucleotide substitution, nonsense | M_D(CCD, ≥25)_ O_H(LV)_ G_AD_ E_G_ S_C-NA_ | Pathogenic | Pathogenic (Id) | Pasotti et al. 2008^9^ |
| c.746G>A, p.Arg249Gln | Exon 4: Single nucleotide substitution, missense | M_E(SA, <25)_ O_H(A)+M_ G_DN_ E_G_ S_C-IV_ | Likely pathogenic | Likely pathogenic (II) | Bonne G et al. 2000^56^ |
| c.751dup, p.Gln251ProfsX3 | Exon 4: Single nucleotide duplication, frameshift and premature termination of translation | M_D(CCD)_ O_H(LV)_ G_NA_ E_G_ S_C-NA_ | Conflicting interpretations of pathogenicity: Likely pathogenic(1);Uncertain significance(1) | Pathogenic (Id) | Arola et al. 2004^16^ |
| c.763delC, p.Gln255Argfs | Exon 4: Single nucleotide deletion, frameshift and premature termination of translation | NA | N/A | Pathogenic (Ic) | https://www.ncbi.nlm.nih.gov/clinvar/variation/48079 |
| c.768G>A, p.Val256Val | Exon 4: Single nucleotide substitution, missense | M_D(CCD)_ O_H(LV)_ G_AD_ E_G_ S_C-NA_ | Likely pathogenic | VUS - not enough evidence | Ito et al. 2014^57^ |
| c.775T>C, p.Tyr259His | Exon 4: Single nucleotide substitution, missense | M_D(SA, CCD, VA, ≥25)_ O_H(LV)_ G_AD_ E_G_ S_C-I_ | Pathogenic | Likely pathogenic (II) | Saga et al. 2009^58^ |
| c.777T>A, p.Tyr259Ter | Exon 4: Single nucleotide substitution, nonsense | M_E(SA, CCD, ↑CK, ≥25)_ O_H_ G_AD_ E_G_ S_C-I_ | Not provided | Pathogenic (Ia) | van Tintelen et al. 2007^12^~ |
| c.780G>C, p.Lys260Asn | Exon 4: Single nucleotide substitution, missense | M_D(CCD)_ O_H(LV)_ G_AD_ E_G_ S_C-NA_ | Pathogenic | Pathogenic (IIIb) | Arbustini et al. 2005^59^ |
| c.781_783delAAG, p.Lys261del | Exon 4: Deletion (3 nucleotides), in-frame deletion | M_D(SA, MVA, ↑CK, <25)_ O_H(LV)+M_ G_AD_ E_G_ S_C-I_ | N/A | Pathogenic (Id) | Felice K et al. 2000^60^ |
| c.799T>C, p.Tyr267His | Exon 4: Single nucleotide substitution, missense | M_D(SA, CCD, VA, ≥25)_ O_H(LV, A)+M_ G_AD_ E_G_ S_C-NA_ | Pathogenic/Likely pathogenic | Pathogenic (IIIb) | Carboni et al. 2012^61^ |
| c.800A>G, p.Tyr267Cys | Exon 4: Single nucleotide substitution, missense | M_D(CCD)_ O_H(LV)+M_ G_AD_ E_G_ S_C-NA_ | Pathogenic | Likely pathogenic (II) | Pasotti et al. 2008^9^ |
| c.810+63C>A | Intron 4: Single nucleotide substitution, not predicted to cause cryptic splicing | M_E(SA)_ O_H_ G_AD_ E_G_ S_C-I_ | Not provided | VUS - not enough evidence | Brauch et al. 2009^36^ |
| c.815_818delinsCCAGAC, p.Asp272AlafsX208 | Exon 5: Deletion + insertion, frameshift and premature termination of translation | M_D(SA, CCD, VA, MVA, ≥25)_ O_H(LV)_ G_AD_ E_G_ S_C-II_ | Not provided | Pathogenic (Ia) | Saga et al. 2009^58^ |
| c.825_832delGCAGTCTG, p.Arg275SerfsX1 | Exon 5: Deletion (8 nucleotides), frameshift and premature termination of translation | NA | Not provided | Pathogenic (Ia) | van Rijsingen et al. 2013^6^ |
| c.832G>A, p.Ala278Thr | Exon 5: Single nucleotide substitution, missense | M_D(SA, VA, MVA, ≥25)_ O_H(LV)+M_ G_AD_ E_G_ S_C-I_ | N/A | Likely pathogenic (II) | Beckman et al. 2010^62^ |
| c.859insC, p.Ala287ArgfsX44 | Exon 5: Single nucleotide insertion, frameshift and premature termination of translation | M_D(SA)_ O_H(LV)_ G_AD_ E_G_ S_C-III_ | N/A | Pathogenic (Ic) | Millat et al. 2009^29^ |
| c.860delC, p.Ala287ValfsX193 | Exon 5: Single nucleotide deletion, frameshift and premature termination of translation | M_D(CCD)_ O_H(LV)_ G_NA_ E_G_ S_C-NA_ | N/A | Pathogenic (Ic) | Karrouz et al. 2009^63^ |
| NM_005572.3: c.863C>G, p.Ala288Gly | Exon 5: Single nucleotide substitution, missense | M_D(SA, ≥25)_ O_H(LV)_ G_AD_ E_G_ S_C-NA_ | Likely pathogenic | Likely pathogenic (II) | https://www.ncbi.nlm.nih.gov/clinvar/variation/48089 |
| c.868G>A, p.Glu290Lys | Exon 5: Single nucleotide substitution, missense | M_D(CCD, MVA, ≥25)_ O_H(LV)+E_ G_AD_ E_G_ S_C-I_ | Conflicting interpretations of pathogenicity | Likely pathogenic (II) | Finsterer et al. 2016^64^ |
| c.871G>A, p.Glu291Lys | Exon 5: Single nucleotide substitution, missense | M_D(CCD, eHF, MVA, <25)_ O_H(LV)_ G_AD_ E_G_ S_C-IV_ | Pathogenic | Likely pathogenic (II) | Perez-Serra et al., 2015^65^ |
| c.892C>T, p.Arg298Cys | Exon 5: Single nucleotide substitution, missense | M_D(SA, CCD, VA, MVA, ↑CK, <25)_ O_H(LV)_ G_AD_ E_G_ S_C-NA_ | Pathogenic | Likely pathogenic (II) | Ben Yao et al. 2007^66^ |
| c.908_909delCT, p.Ser303CysfsX27 | Exon 5: Deletion (2 nucleotides), frameshift and premature termination of translation | M_D(SA, CCD, VA, MVA)_ O_H(LV)+M_ G_AD_ E_G_ S_C-NA_ | Pathogenic: Dilated cardiomyopathy 1A, Dilated cardiomyopathy 1S, Charcot-Marie-Tooth disease, type 2 | Pathogenic (Ia) | Antoniades et al. 2007^67^ |
| c.936G>C, p.Gln312His | Exon 5: Single nucleotide substitution, missense | M_D(CCD, eHF)_ O_H(LV)+M_ G_AD_ E_G_ S_C-NA_ | N/A | Likely pathogenic (II) | Ben Yao et al. 2005^68^ |
| c.936+1delG | Intron 5: Single nucleotide deletion, predicted abnormal splicing | M_D_ O_H(LV)_ G_AD_ E_G_ S_C-NA_ | N/A | VUS - not enough evidence | van Rijsingen et al. 2013^6^, |
| c.936+2T>G | Intron 5: Single nucleotide substitution, predicted abnormal splicing | M_D_ O_H(LV)_ G_AD_ E_G_ S_C-NA_ | N/A | VUS - not enough evidence | van Spaendonck-Zwarts et al. 2013^69^ |
| c.936+1G>T | Intron 5: Single nucleotide substitution, predicted abnormal splicing | M_D(CCD, VA, MVA, ≥25)_ O_H(LV)_ G_AD_ E_G_ S_C-III_ | Not provided | Likely pathogenic (II) | Arbustini et al. 2007^23^ |
| c.937-11C>G | Intron 5: Single nucleotide substitution, predicted abnormal splicing | M_D(SA, CCD, VA, ≥25)_ O_H(LV, LVRV, A)+M_ G_AD_ E_G_ S_C-NA_ | N/A | Likely pathogenic (II) | Carboni et al 2011^70^ |
| c.937-46A>G | Intron 5: Single nucleotide substitution, predicted abnormal splicing | M_D(SA, eHF)_ O_H_ G_DN_ E_G_ S_C-NA_ | N/A | VUS - not enough evidence | Brauch et al. 2009^36^, Banerjee A et al. 2015^71^ |
| c.949G>A, p.Glu317Lys | Exon 6: Single nucleotide substitution, missense | M_D(SA, CCD, eHF, ≥25)_ O_H(LV)_ G_AD_ E_G_ S_C-NA_ | Pathogenic/Likely pathogenic | Likely pathogenic (II) | Arbustini et al. 2002^31^ |
| c.952G>A, p.Ala318Thr | Exon 6: Single nucleotide substitution, missense | M_D(SA, CCD, eHF, VA, ≥25)_ O_H(LV)_ G_AD_ E_G_ S_C-IV_ | Not provided | Likely pathogenic (II) | Parks et al. 2009^32^ |
| NM_005572.3:c.959delT, p.Arg321GlufsX159 | Exon 6: Single nucleotide deletion, frameshift and premature termination of translation | M_D(SA, CCD, eHF, VA, MVA, ↑CK, <25)_ O_H(LV)+M_ G_AD_ E_G_ S_C-III_ | Pathogenic | Pathogenic (Ia) | Brodsky et al. 2000^44^ |
| c.961C>T, p.Arg321Ter | Exon 6: Single nucleotide substitution, nonsense | M_D(CCD, VA, <25)_ O_H_ G_AD_ E_G_ S_C-NA_ | Pathogenic | Likely pathogenic (II) | Hasselberg et al. 2014^72^ |
| c.992G>A, p.Arg331Gln | Exon 6: Single nucleotide substitution, missense | M_D(≥25)_ O_H_ G_AD_ E_G_ S_C-NA_ | Pathogenic | Likely pathogenic (II) | Benedetti et al. 2007^73^, Hoorntje et al. 2017^74^ |
| c.1001G>A, p.Ser334Asn | Exon 6: Single nucleotide substitution, missense | NA | Pathogenic/Likely pathogenic | VUS - not enough evidence | van Spaendonck-Zwarts et al. 2013^69^ |
| c.1003C>T, p.Arg335Trp§ | Exon 6: Single nucleotide substitution, missense | M_D(SA, CCD, eHF, VA, ≥25)_ O_H(LV)_ G_AD_ E_G_ S_C-IV_ | N/A | Likely pathogenic (II) | Stallmeyer et al. 2012^75^ |
| c.1004G>A, p.Arg335Gln | Exon 6: Single nucleotide substitution, missense | M_D(CCD)_ O_H(LV)_ G_DN_ E_G_ S_C-NA_ | Pathogenic/Likely pathogenic | VUS - not enough evidence | Ehlermann et al. 2009^76^ |
| c.1039G>A, p.Glu347Lys | Exon 6: Single nucleotide substitution, missense | M_D(SA, CCD, ↑CK, MVA, ≥25)_ O_H(LV)+M_ G_AD_ E_G_ S_C-NA_ | Uncertain significance | Likely pathogenic (II) | Vytopil et al. 2003^77^ Bollati et al. 2012^78^ |
| c.1044G>T, p.Met348Ile | Exon 6: Single nucleotide substitution, missense | M_D(CCD)_ O_H(LV)_ G_AD_ E_G_ S_C-NA_ | Likely pathogenic | Likely pathogenic (II) | Meinke et al. 2011^79^ |
| c.1045C>T, p.Arg349Trp | Exon 6: Single nucleotide substitution, missense | M_D(SA, CCD, eHF, ↑CK, <25)_ O_H(LV)+M+L+E_ G_AD_ E_G_ S_C-IV_ | Not provided | Likely pathogenic (II) | van Tintelen et al. 2007^12^~ |
| c.1046G>T, p.Arg349Leu | Exon 6: Single nucleotide substitution, missense | M_D(SA, CCD, eHF, <25)_ O_H(LV)_ G_AD_ E_G_ S_C-IV_ | Pathogenic | Likely pathogenic (II) | Hermida-Prieto et al. 2004^80^ |
| c.1048G>C, p.Ala350Pro | Exon 6: Single nucleotide substitution, missense | M_D(SA, CCD, VA, ↑CK, <25)_ O_H(LV)+M_ G_AD_ E_G_ S_C-II_ | Not provided | Likely pathogenic (II) | Rudenskaya et al. 2008^17^ |
| c.1057C>A, p.Gln353Lys | Exon 6: Single nucleotide substitution, missense | M_D(VA, ↑CK, ≥25)_ O_H(LV)+M_ G_AD_ E_G_ S_C-IV_ | Not provided | Pathogenic (II) | Gupta et al. 2010^46^ |
| c.1063C>T, p.Gln355Ter | Exon 6: Single nucleotide substitution, nonsense | M_D(SA, CCD, eHF, ≥25)_ O_H(LV)_ G_AD_ E_G_ S_C-IV_ | Not provided | Likely pathogenic (II) | Pethig et al. 2005^11^ |
| c.1069G>C, p.Asp357His | Exon 6: Single nucleotide substitution, missense | M_D(CCD, MVA)_ O_H(LV)_ G_AD_ E_G_ S_C-NA_ | Pathogenic | Likely pathogenic (II) | Fujimori et al. 2008^81^ |
| c.1070A>C, p.Asp357Ala | Exon 6: Single nucleotide substitution, missense | M_D(SA, CCD, eHF, VA, MVA, <25)_ O_H(LV)_ G_AD_ E_G_ S_C-IV_ | Not provided | VUS - not enough evidence | Stallmeyer et al. 2012^75^ |
| c.1072G>A, p.Glu358Lys | Exon 6: Single nucleotide substitution, missense | M_D(CCD)_ O_H(LV)+M_ G_NA_ E_G_ S_C-NA_ | N/A | Likely pathogenic (II) | Dittmer et al. 2014^82^ |
| c.1072G>T, p.Glu358Ter | Exon 6: Single nucleotide substitution, nonsense | M_E(CCD, VA, MVA, ≥25)_ O_H(LV)_ G_AD_ E_G_ S_C-I_ | Pathogenic | Pathogenic (Ia) | De Backer et al. 2010^83^ |
| c.1085delT, p.Leu363TrpfsX117 | Exon 6: Single nucleotide deletion, frameshift and premature termination of translation | M_D(SA, CCD, VA, eHF, ≥25)_ O_H(LV)_ G_AD_ E_G_ S_C-IV_ | Not provided | Pathogenic (Ia) | Kärkkäinen et al. 2006^37^ |
| c.1112_1115dupTGGA, p.Glu372Aspfs | Exon 6: Duplication (4 nucleotides), frameshift and premature termination of translation | M_D(SA, CCD, MVA, ≥25)_ O_H(LV)_ G_AD_ E_G_ S_C-NA_ | Likely pathogenic | Pathogenic (Ic) | Parks et al. 2009^32^ |
| c.1114delG, p.Glu372ArgfsX108 | Exon 6: Single nucleotide deletion, frameshift and premature termination of translation | M_D(SA, CCD, eHF, VA, ≥25)_ O_H(LV)_ G_AD_ E_G_ S_C-IV_ | Pathogenic | Likely pathogenic (II) | Parks et al. 2009^32^ |
| c.1128C>A, p.Tyr376Ter | Exon 6: Single nucleotide substitution, nonsense | NA | Pathogenic/Likely pathogenic | Likely pathogenic (II) | van Rijsingen et al. 2013^6^ |
| c.1129G>A, p.Arg377Gly (erroneously reported as p.Arg377His)) | Exon 6: Single nucleotide substitution, missense | M_D(SA, CCD, ≥25)_ O_H(LV)_ G_AD_ E_G_ S_C-NA_ | Not provided | Likely pathogenic (II) | Perrot et al. 2006^39^ |
| c.1129C>T, p.Arg377Cys | Exon 6: Single nucleotide substitution, missense | M_D(eHF, <25)_ O_H(LV)+M_ G_AD_ E_G_ S_C-IV_ | N/A | Likely pathogenic (II) | Komaki et al. 2011^84^, Anselme et al. 2013^85^, Kumar et al. 2016^24^ |
| c.1130G>A, p.Arg377His | Exon 6: Single nucleotide substitution, missense | M_D(SA, CCD, eHF, VA, MVA, <25)_ O_H(LV)+M_ G_AD_ E_G_ S_C-IV_ | N/A | Likely pathogenic (II) | Charniot et al. 2003^86^ |
| c.1130G>T, p.Arg377Leu | Exon 6: Single nucleotide substitution, missense | M_E(SA, CCD, MVA, ↑CK, <25)_ O_H(LV)+M_ G_AD_ E_G_ S_C-NA_ | Pathogenic/Likely pathogenic | Likely pathogenic (II) | van Tintelen et al. 2007^12^~ |
| c.1142delA, p.Glu381GlyfsX99 | Exon 6: Single nucleotide deletion, frameshift and premature termination of translation | M_D(eHF, VA)_ O_H(LV)_ G_NA_ E_G_ S_C-IV_ | Pathogenic | Pathogenic (Ic) | Kumar et al. 2016^24^ |
| c.1146C>T, p.Gly382Gly | Exon 6: Single nucleotide substitution, synonymous; predicted creation of new splice donor site resulting in deletion of the last 13 nucleotides of exon 6 and frameshift | M_D(MVA)_ O_H_ G_NA_ E_G_ S_C-NA_ | Uncertain significance | Pathogenic (Ic) | Benedetti et al. 2007^73^, Quenin et al. 2017^87^ |
| c.1150G>T, p.Glu384Ter | Exon 6: Single nucleotide substitution, nonsense | M_D_ O_H(LV)_ G_NA_ E_G_ S_C-NA_ | N/A | Pathogenic (Ic) | Hirlte-Lewis et al. 2013^43^ |
| c.1157G>C, p.Arg386Thr | Exon 6: Single nucleotide substitution, missense | M_D(CCD, VA, ≥25)_ O_H(LV)+M_ G_AD_ E_G_ S_C-NA_ | Pathogenic/Likely pathogenic | Likely pathogenic (II) | Bonne G et al. 2000^56^ |
| c.1157+1G>A | Intron 6: Single nucleotide substitution, predicted abnormal splicing | M_D(eHF, VA, MVA, <25)_ O_H(LV)_ G_AD_ E_G_ S_C-IV_ | N/A | VUS - not enough evidence | Pasotti et al. 2008^9^ |
| c.1157+1G>T, p.Arg386SerfsX21 | Intron 6: Single nucleotide substitution, frameshift and premature termination of translation | M_D(eHF, VA, MVA, ≥25)_ O_H(LV)_ G_AD_ E_G_ S_C-IV_ | Pathogenic | Pathogenic (Ic) | Stallmeyer et al. 2012^75^ |
| c.1158-44C>T | Intron 6: Single nucleotide substitution, predicted abnormal splicing | NA | Not provided | VUS - not enough evidence | Fokkema et al. 2005^28^ |
| c.1163G>A, p.Arg388His | Exon 7: Single nucleotide substitution, missense | M_D(SA, CCD, eHF, VA, MVA, <25)_ O_H(LV)_ G_AD_ E_G_ S_C-IV_ | Pathogenic | Likely pathogenic (II) | Parks et al. 2009^32^ |
| c.1173dup, p.Ser392GlnfsX34 | Exon 7: Single nucleotide duplication, frameshift and premature termination of translation | M_D(eHF, VA)_ O_H(LV)_ G_NA_ E_G_ S_C-IV_ | Benign | VUS - not enough evidence | Kumar et al. 2016^24^ |
| c.1189C>T, p.Arg397Cys | Exon 7: Single nucleotide substitution, missense | M_D_ O_H(LV)_ G_NA_ E_G_ S_C-NA_ | Not provided | VUS - not enough evidence | Narula et al. 2012, van Rijsingen et al. 2013^6^ |
| c.1195C>T, p.Arg399Cys | Exon 7: Single nucleotide substitution, missense | M_D(CCD, eHF, VA, MVA, <25)_ O_H(LV)_ G_AD_ E_G_ S_C-IV_ | N/A | Likely pathogenic (II) | Parks et al. 2009^32^ |
| c.1243G>A, p.Val415Ile | Exon 7: Single nucleotide substitution, missense | M_E(SA)_ O_H_ G_DN_ E_G_ S_C-I_ | Uncertain significance | VUS - not enough evidence | Brauch et al. 2009^36^ |
| c.1294C>T, p.Gln432Ter | Exon 7: Single nucleotide substitution, nonsense | M_D(SA, CCD, VA, ≥25)_ O_H(LV)_ G_AD_ E_G_ S_C-NA_ | Conflicting interpretations of pathogenicity | Pathogenic (Ia) | Møller et al. 2009^88^ |
| c.1303C>T, p.Arg435Cys | Exon 7: Single nucleotide substitution, missense | M_D(eHF, <25)_ O_H(LV)+M_ G_AD_ E_G_ S_C-IV_ | Uncertain significance | Likely pathogenic (II) | Vytopil et al. 2003^77^ |
| c.1307_1308insGCAC, p.Ser437HisfsX2 | Exon 7: Duplication (4 nucleotides), frameshift and premature termination of translation | M_D(SA, CCD, eHF, VA, MVA, <25)_ O_H(LV)_ G_AD_ E_G_ S_C-IV_ | Pathogenic/Likely pathogenic | Pathogenic (Ia) | Parks et al. 2009^32^ |
| c.1318G>A, p.Val440Met | Exon 7: Single nucleotide substitution, missense | M_D(CCD)_ O_H(LV)_ G_NA_ E_G_ S_C-NA_ | Conflicting interpretations of pathogenicity | Likely pathogenic (II) | Dittmer et al. 2014^82^ |
| c.1322C>T, p.Ala441Val | Exon 7: Single nucleotide substitution, missense | M_D_ O_H_ G_NA_ E_G_ S_C-NA_ | Pathogenic | VUS - not enough evidence | Perrot et al. 2010^89^ |
| c.1380+1G>T | Intron 7: Single nucleotide substitution, predicted abnormal splicing | M_D(VA)_ O_H(LV)_ G_NA_ E_G_ S_C-NA_ | Conflicting interpretations of pathogenicity | Likely pathogenic (II) | Kumar et al. 2016^24^ |
| c.1380+1G>A | Intron 7: Single nucleotide substitution, predicted abnormal splicing | M_D(SA, CCD, MVA, ≥25)_ O_H(LV)_ G_AD_ E_G_ S_C-NA_ | N/A | Likely pathogenic (II) | van Tintelen et al. 2007^12^ |
| c.1381G>T, p.Asp461Tyr | Exon 8: Single nucleotide substitution, missense | M_D(CCD)_ O_H(LV)_ G_AD_ E_G_ S_C-NA_ | N/A | Likely pathogenic (II) | Scharner et al 2013^90^ |
| c.1397delA, p.Asn466IlefsX14 | Exon 8: Single nucleotide deletion, frameshift and premature termination of translation | M_D_ O_H_ G_NA_ E_G_ S_C-NA_ | Pathogenic | Pathogenic (Ia) | Genschel et al. 2000^91^ |
| c.1412G>A, p.Arg471His | Exon 8: Single nucleotide substitution, missense | M_D(SA, eHF, VA, MVA, <25)_ O_H(LV)_ G_AD_ E_G_ S_C-IV_ | Not provided | Likely pathogenic (II) | Parks et al. 2009^32^ |
| c.1424_1425insAGA, p.Gly474_Asp475insGlu | Exon 8: Duplication (3 nucleotides), inframe insertion of single amino acid | M_D(VA, ≥25)_ O_H(LV)_ G_AD_ E_G_ S_C-NA_ | Not provided | Likely pathogenic (II) | Parks et al. 2009^32^ |
| c.1443C>G, p.Tyr481Ter | Exon 8: Single nucleotide substitution, nonsense | M_D(SA, CCD, eHF, <25)_ O_H(LV)_ G_AD_ E_G_ S_C-IV_ | Conflicting interpretations of pathogenicity | Pathogenic (Ia) | Sylvius et al. 2005^50^ |
| c.1462A>C, p.Thr488Pro | Exon 8: Single nucleotide substitution, missense | M_E(SA, ≥25)_ O_H_ G_NA_ E_G_ S_C-I_ | N/A | VUS - not enough evidence | Brauch et al. 2010^36^ |
| c.1489-1G>T, p.Ile497_Glu536del | Intron 8: Single nucleotide substitution, inframe deletion of 40 amino acids | M_D(SA, CCD, VA, ↑CK, <25)_ O_H(LV)+M_ G_AD_ E_G_ S_C-NA_ | Not provided | VUS - not enough evidence | Stallmeyer et al. 2012^75^ |
| c.1489-2A>G | Intron 8: Single nucleotide substitution, predicted abnormal splicing | NA | Not provided | VUS - not enough evidence | van Rijsingen et al. 2013^6^ |
| c.1492T>A, p.Trp498Arg | Exon 9: Single nucleotide substitution, missense | M_D(CCD, VA, MVA, ↑CK, <40)_ O_H(LV)+M_ G_AD_ E_G_ S_C-NA_ | N/A | Likely pathogenic (II) | Pasotti et al. 2008^9^ |
| c.1493delG, p.Ala499LeufsX49 | Exon 9: Single nucleotide deletion, frameshift and premature termination of translation | M_D(SA, VA, ≥25)_ O_H(LV)_ G_AD_ E_G_ S_C-NA_ | Likely pathogenic | Pathogenic (Ia) | Kärkkäinen et al. 2006^37^ |
| c.1496delC, p.Ala499ValfsX49 | Exon 9 Single nucleotide deletion, frameshift and premature termination of translation | M_D(SA, CCD, eHF, ↑CK, ≥25)_ O_H(LVRV)+N+M_ G_AD_ E_G_ S_C-IV_ | Not provided | Pathogenic (Ia) | Duparc et al. 2009^92^ |
| c.1512–1513insAG, p.Thr505ArgfsX44 | Exon 9: Insertion (2 nucleotides), frameshift and premature termination of translation | M_D(SA, CCD, eHF, VA, MVA, ≥25)_ O_H(LV)_ G_AD_ E_G_ S_C-IV_ | Not provided | Pathogenic (Ia) | van Tintelen et al. 2007^12^ |
| c.1526insA, p.Thr510TyrfsX42 | Exon 9: Single nucleotide insertion, frameshift and premature termination of translation | M_D(SA, CCD, eHF, MVA, <25)_ O_H(LV)_ G_AD_ E_G_ S_C-IV_ | Not provided | Pathogenic (Ia) | Chen et al. 2013^93^ |
| c.1526insC, p.Thr510TyrfsX41 | Exon 9: Single nucleotide insertion, frameshift and premature termination of translation | M_D(SA, CCD, CK, eHF, VA, MVA, <25)_ O_H(LV)+M_ G_AD_ E_G_ S_C-IV_ | Not provided | Pathogenic (Ia) | Saj et al. 2013^94^, van Rijsingen et al. 2013^6^ |
| c.1542G>A, p.Trp514X | Exon 9: Single nucleotide substitution, nonsense | NA | N/A | Pathogenic (Ic) | van Rijsingen et al. 2013^6^ |
| c.1549C>T, p.Gln517Ter | Exon 9: Single nucleotide substitution, nonsense | M_D(SA, CCD, VA, MVA, ≥25)_ O_H(LV)+M_ G_AD_ E_G_ S_C-NA_ | Pathogenic/Likely pathogenic | Pathogenic (Ic) | Stallmeyer et al. 2012^75^ |
| c.1560G>A, p.Trp520Ter | Exon 9: Single nucleotide substitution, nonsense | M_D(CCD, ≥25)_ O_H(LV)_ G_AD_ E_G_ S_C-NA_ | N/A | Likely pathogenic (II) | Stallmeyer et al. 2012^75^ |
| c.1567G>A, p.Gly523Arg | Exon 9: Single nucleotide substitution, missense | M_D_ O_H(LV)_ G_AD_ E_G_ S_C-NA_ | N/A | Likely pathogenic (II) | Millat et al. 2009^29^ |
| c.1576_1579dup, p.Arg527ProfsX26 (reported as c.1579_1580insCTGC, p.Ile527fsX23) | Exon 9: Duplication (4 nucleotides), frameshift and premature termination of translation | M_D(CCD, eHF, MVA, <40)_ O_H(LV)_ G_AD_ E_G_ S_C-IV_ | Pathogenic | Pathogenic (Ia) | Pasotti et al. 2008^9^ |
| c.1580G>C, p.Arg527Pro | Exon 9: Single nucleotide substitution, missense | M_D(CCD)_ O_H(LV)_ G_NA_ E_G_ S_C-NA_ | Uncertain significance | Pathogenic (II) | Dittmer et al. 2014^82^ |
| c.1608+4A>G | Intron 9: Single nucleotide substitution, predicted abnormal splicing | M_D(eHF)_ O_H(LV)_ G_AD_ E_G_ S_C-IV_ | N/A | VUS - not enough evidence | te Rijdt et al. 2017^95^, van Spaendonck-Zwarts et al. 2013^69^ |
| c.1608+14G>A | Intron 9: Single nucleotide substitution, predicted abnormal splicing | NA | Pathogenic | Likely pathogenic (V) | van Rijsingen et al. 2013^6^ |
| c.1609-12T>G, p.Glu536fsX14 | Intron 9: Single nucleotide substitution, frameshift and premature termination of translation | M_D(SA, CCD, VA, MVA, ↑CK, ≥25)_ O_H(LV)+M_ G_AD_ E_G_ S_C-NA_ | N/A | Pathogenic (Ic) | Renou et al. 2008^96^ |
| c.1609-3C>G | Intron 9: Single nucleotide substitution, predicted abnormal splicing | M_D(CCD, eHF, VA, MVA)_ O_H(LV)+M_ G_AD_ E_G_ S_C-IV_ | N/A | Likely pathogenic (V) | Chrestian N et al. 2008^97^, Fokkema et al. 2005^28^ |
| c.1621C>T, p.Arg541Cys | Exon 10: Single nucleotide substitution, missense | M_D(SA, CCD)_ O_H(LV)_ G_NA_ E_G_ S_C-NA_ | Pathogenic | Likely pathogenic (II) | Dittmer et al. 2014^82^ |
| c.1621C>G, p.Arg541Gly | Exon 10: Single nucleotide substitution, missense | M_D(CCD)_ O_H(LV)_ G_NA_ E_G_ S_C-NA_ | Pathogenic/Likely pathogenic | Likely pathogenic (II) | Dittmer et al. 2014^82^ |
| c.1621C>A, p.Arg541Ser | Exon 10: Single nucleotide substitution, missense | M_D(SA, eHF, ↑CK, <25)_ O_H(LV)+M_ G_AD_ E_G_ S_C-IV_ | Pathogenic | Likely pathogenic (II) | Sylvius et al. 2005^50^ |
| c.1622G>A, p.Arg541His | Exon 10: Single nucleotide substitution, missense | M_D(SA, CCD, VA, <25)_ O_H(LV)_ G_AD_ E_G_ S_C-II_ | Pathogenic | Likely pathogenic (II) | Rudenskaya et al. 2008^17^ |
| c.1622G>C, p.Arg541Pro (erroneously reported as p.Arg541Lys) | Exon 10: Single nucleotide substitution, missense | M_D(CCD, eHF, VA, ↑CK, <25)_ O_H(LV)+M_ G_DN_ E_G_ S_C-IV_ | Not provided | Likely pathogenic (II) | van Tintelen et al. 2007^12^ |
| c.1634G>A, p.Arg545His | Exon 10: Single nucleotide substitution, missense | M_E(CCD)_ O_H(LV)+M_ G_DN_ E_G_ S_C-I_ | Conflicting interpretations of pathogenicity | VUS - not enough evidence | van Rijsingen et al. 2013^6^, Olaopa et al. 2018^98^ |
| c.1657G>A, p.Asp553Asn | Exon 10: Single nucleotide substitution, missense | M_D(VA)_ O_H(LV)_ G_NA_ E_G_ S_C-NA_ | Likely pathogenic | VUS - not enough evidence | Kumar et al. 2016^24^ |
| c.1698+83G>A | Intron 10: Single nucleotide substitution, predicted abnormal splicing | M_D(CCD)_ O_H(LV)_ G_AD_ E_G_ S_C-NA_ | Conflicting interpretations of pathogenicity | VUS - not enough evidence | Benedetti et al. 2004^99^ |
| NM_005572.3:c.1713C>A, p.Ser571Arg (erroneously reported as c.1711C>A, p.Arg571Ser) | Exon 11: Single nucleotide substitution, missense | M_D(SA, CCD, ↑CK, ≥25)_ O_H(LV)_ G_AD_ E_G_ S_C-NA_ | Uncertain significance | VUS - not enough evidence | Fatkin et al. 1999^21^ |
| c.1714insCTGC, p.Ser572LeufsX8 | Exon 11: Insertion (4 nucleotides), frameshift and premature termination of translation | M_D(CCD, eHF, VA, ≥25)_ O_H(LV)_ G_AD_ E_G_ S_C-IV_ | N/A | Pathogenic (Id) | Arbustini et al. 2002^31^ |
| c.1718C>T, p.Ser573Leu | Exon 11: Single nucleotide substitution, missense | M_D(VA, ≥25)_ O_H(LV)_ G_DN_ E_G_ S_C-III_ | Pathogenic | Pathogenic (IIIb) | Taylor et al. 2003^27^ |
| c.1839_1840insC, p.Arg614GlyfsX89 | Exon 11: Single nucleotide insertion, frameshift and premature termination of translation | NA | N/A | Pathogenic (Id) | van Rijsingen et al. 2013^6^ |
| c.1851C>T, p.Ala617Ala | Exon 11: Single nucleotide substitution, missense | NA | Conflicting interpretations of pathogenicity | VUS - not enough evidence | Fokkema et al. 2005^28^ |
| c.1904G>A, p.Gly635Asp | Exon 11: Single nucleotide substitution, missense | M_D(CCD)_ O_H(LV)_ G_NA_ E_G_ S_C-NA_ | N/A | Likely pathogenic (II) | Dittmer et al. 2014^82^ |
| c.1912G>A, p.Gly638Arg | Exon 11: Single nucleotide substitution, missense | NA | Conflicting interpretations of pathogenicity | VUS - not enough evidence | van Rijsingen et al. 2013^6^, Pugh et al. 2014^100^ |
| c.1930C>T, p.Arg644Cys | Exon 11: Single nucleotide substitution, missense | M_D_ O_H(LV)_ G_AD_ E_G_ S_C-NA_ | Not provided | Likely pathogenic (III) | Genschel et al. 2001^101^ |
| c.1960C>T, p.Arg654Ter | Exon 11: Single nucleotide substitution, nonsense | M_D(SA, CCD, VA, MVA, ≥25)_ O_H(LV)_ G_AD_ E_G_ S_C-NA_ | Conflicting interpretations of pathogenicity | Likely pathogenic (II) | Parks et al. 2009^32^ |
| c.1975dup, p.Gln659delinsSer | Exon 12: Single nucleotide duplication, inframe deletion and insertion of single amino acid | M_D(VA, MVA)_ O_H(LV)_ G_NA_ E_G_ S_C-NA_ | Conflicting interpretations of pathogenicity | VUS - not enough evidence | Moller et al. 2010^102^ |

* Mutations are ordered by exonic location.

∞ The summative (overall) MOGE(S) class for each mutation was derived using available data reported for the mutation-positive proband or affected family members in the published literature (of which at least 1 citation is reproduced here). If phenotypic heterogeneity existed between reported probands/families, the descriptor was taken as the most severe manifestation reported or the youngest reported age of presentation. Mutations with missing phenotype information are labeled NA. **MOGE(S)** nosology^103^ used throughout this table is as follows:

**M, morphofunctional phenotype with main descriptors**:

**D**, dilated cardiomyopathy

**E**, early phenotype not fulfilling criteria for DCM (e.g. mild LV dilatation with preserved/low-normal systolic function or normal LV cavity size and function but other clinical features consistent with subclinical cardiac disease e.g. CCD/Arrhythmia/Atrial dilatation)

**NA**, not available

To ‘**D**’ or ‘**E**’ we have added the following **key clinical red flags**:

**SA**, supraventricular arrhythmia

**CCD**, cardiac conduction system disease

**eHF**, end-stage heart failure defined as heart transplantation or death from end-stage heart failure

**VA**, ventricular arrhythmia defined as any type of non-life threatening VA including ventricular ectopy

**MVA,** malignant ventricular arrhythmia defined as the potentially life-threatening VA, sudden cardiac death, resuscitation or appropriate defibrillator therapy

**↑CK,** elevated creatine phosphokinase

**<25** or **≥25**, to differentiate between mutations of early versus late phenotypic penetrance, considering the earliest reported age of phenotypic expression in the proband or affected family members. Where age of phenotypic penetrance was not indicated by authors in the cited work the denomination is omitted from the MOGE(S). Where authors only reported ages as </≥30 or </≥40 a similar cut off was used. If age was not reported it is omitted from the table.

**O, organ system involvement with main descriptors:**

**H**, heart

**M**, skeletal muscle (any report of even mild symptomatic myopathy is counted as skeletal muscle involvement but isolated high CPK with no symptoms or confirmatory muscle tests is not)

**N**, nervous

**L**, lipid (lipodystrophy)

**E**, endocrine (e.g. insulin abnormalities)

**NA**, not available

To ‘**H**’ we have added the following denominators:

**LV**, left ventricle involvement

**RV**, right ventricle involvement

**LRV**, biventricular involvement

**A**, atrial involvement

**G, genetic inheritance pattern with main descriptors:**

**AD**, autosomal dominant where the cited publication suggests other likely affected family members

**DN**, de novo disease-causing mutation where the cited publication includes indicates a patient who is the uniquely affected member of the family, and in the absence of a family history of CM

**NA,** not available

**E, etiology with main descriptor:**

**G**, genetic (applies to all cases here)

**S, functional status with main descriptor:**

**C_I-IV_**, as the most severe New York Heart Association Functional class (NYHA I-IV) reported in the patient or in the affected family members in the cited paper. If NYHA functional class was not specified in the cited paper but reported phenotype included **eHF**, then the most severe NYHA functional class was assumed to be class IV.

**NA**, not available

**Note-1:** *LMNA* mutations linked primarily to LGMD, EDMD, FPLD or progeroid syndromes, where DCM is reported as an associated, but not the primary clinical manifestation, have not been included in this table.

*ACMG, American College of Medical Genetics and Genomics; EDMD, Emery-Dreifuss muscular dystrophy; FPLD, Familial partial lipodystrophy, Dunnigan variety; LAP2-α, Lamin associated protein; LGMD, Limb-girdle muscular dystrophy; NLS, nuclear localisation signal.*

**SUPPLEMENTARY TABLE 2. Solutions from the various clustering algorithms.** All 7 cluster algorithms consistently generate a 2-branch dendrogram with 4 contained subclusters.

| **Cluster Algorithm** | **Output** | **Cophenetic Correlation with method ‘Ward’** |
| --- | --- | --- |
| Ward* | Dendrogram with 2 branches and 176 members total, at height 90.3 | – |
| Single | Dendrogram with 2 branches and 176 members total, at height 2.1 | 0.84 |
| Complete | Dendrogram with 2 branches and 176 members total, at height 4.0 | 0.90 |
| Average | Dendrogram with 2 branches and 176 members total, at height 3.1 | 0.91 |
| Mcquitty | Dendrogram with 2 branches and 176 members total, at height 3.1 | 0.77 |
| Median^#^ | Dendrogram with 2 branches and 176 members total, at height 1.2 | 0.43 |
| Centroid^#^ | Dendrogram with 2 branches and 176 members total, at height 1.6 | 0.46 |

Seven cluster objects, including our chosen method (*) were tested using ‘hclust’ chained together as a single dendlist object.

# Cluster methods ‘Median’ and ‘Centroid’ are less meaningful when applied to ordinal categorical data like ours, where use of the other methods is more appropriate.

**SUPPLEMENTARY TABLE 3. Cluster conformity test results.** When data was cut to k=4 clusters using the Fowlkes-Mallows Index (excluding methods ‘Median’ and ‘Centroid’), all cluster solutions retained good agreement with the chosen method ‘Ward’ (*, FM indices > 0.7).

| **Algorithm** | **Ward*** | **Single** | **Complete** | **Average** | **McQuitty** |
| --- | --- | --- | --- | --- | --- |
| **Ward*** | 1.00 | NA | 1.00 | 0.74 | 0.87 |
| **Single** | NA | 1.00 | NA | NA | NA |
| **Complete** | 1.00 | NA | 1.00 | 0.74 | 0.87 |
| **Average** | 0.74 | NA | 0.74 | 1.00 | 0.84 |
| **McQuitty** | 0.87 | NA | 0.87 | 0.84 | 1.00 |

**REFERENCES**

1. Schwarz JM, Rodelsperger C, Schuelke M, Seelow D. MutationTaster evaluates disease-causing potential of sequence alterations. Nat. Methods. 2010;7:575–576.

2. Adzhubei IA, Schmidt S, Peshkin L, Ramensky VE, Gerasimova A, Bork P, Kondrashov AS, Sunyaev SR. A method and server for predicting damaging missense mutations. Nat. Methods. 2010;7:248–249.

3. Kumar P, Henikoff S, Ng PC. Predicting the effects of coding non-synonymous variants on protein function using the SIFT algorithm. *Nat Protoc*. 2009;4:1073–1081.

4. Gonzalez-Perez A, Lopez-Bigas N. Improving the assessment of the outcome of nonsynonymous SNVs with a consensus deleteriousness score, Condel. *Am J Hum Genet*. 2011;88:440–449.

5. Van Tintelen JP, Tio RA, Kerstjens-Frederikse WS, van Berlo JH, Boven LG, Suurmeijer AJH, White SJ, den Dunnen JT, te Meerman GJ, Vos YJ, van der Hout AH, Osinga J, van den Berg MP, van Veldhuisen DJ, Buys CHCM, Hofstra RMW, Pinto YM. Severe myocardial fibrosis caused by a deletion of the 5’ end of the lamin A/C gene. *J Am Coll Cardiol*. 2007;49:2430–2439.

6. Van Rijsingen IAW, Nannenberg EA, Arbustini E, Elliott PM, Mogensen J, Hermans-van Ast JF, van der Kooi AJ, van Tintelen JP, van den Berg MP, Grasso M, Serio A, Jenkins S, Rowland C, Richard P, Wilde AAM, Perrot A, Pankuweit S, Zwinderman AH, Charron P, Christiaans I, Pinto YM. Gender-specific differences in major cardiac events and mortality in lamin A/C mutation carriers. *Eur J Heart Fail*. 2013;15:376–384.

7. Bécane H, Bonne G, Varnous S, Ortega V, Hammouda ELH, Lavergne T, Fardeau M, Weber S, Schwartz K, Duboc D. High incidence of sudden death with conduction system and myocardial disease due to lamins A and C gene mutation. *Pacing Clin Electrophysiol*. 2000;23:1661–1666.

8. Sébillon P, Bouchier C, Bidot LD, Bonne G, Ahamed K, Charron P, Drouin-Garraud V, Millaire A, Desrumeaux G, Benaïche A, Charniot J, Schwartz K, Villard E, Komajda M. Expanding the phenotype of mutations in dilated cardiomyopathy and functional consequences of these LMNA mutations. *J Med Genet*. 2003;40:560–567.

9. Pasotti M, Klersy C, Pilotto A, Marziliano N, Rapezzi C, Serio A, Mannarino S, Gambarin F, Favalli V, Grasso M, Agozzino M, Campana C, Gavazzi A, Febo O, Marini M, Landolina M, Mortara A, Piccolo G, Viganò M, Tavazzi L, Arbustini E. Long-term outcome and risk stratification in dilated cardiolaminopathies. *J Am Coll Cardiol*. 2008;52:1250–1260.

10. Ho JCY, Zhou T, Lai WH, Huang Y, Chan YC, Li X, Wong NLY, Li Y, Au KW, Guo D, Xu J, Siu CW, Pei D, Tse HF, Esteban MA. Generation of induced pluripotent stem cell lines from 3 distinct laminopathies bearing heterogeneous mutations in lamin A/C. *Aging*. 2011;3:380–390.

11. Pethig K, Genschel J, Peters T, Wilhelmi M, Flemming P, Lochs H, Haverich A, Schmidt HH-J. LMNA mutations in cardiac transplant recipients. *Cardiology*. 2005;103:57–62.

12. Van Tintelen JP, Hofstra RMW, Katerberg H, Rossenbacker T, Wiesfeld ACP, du Marchie Sarvaas GJ, Wilde AAM, van Langen IM, Nannenberg EA, van der Kooi AJ, Kraak M, van Gelder IC, van Veldhuisen DJ, Vos Y, van den Berg MP. High yield of LMNA mutations in patients with dilated cardiomyopathy and/or conduction disease referred to cardiogenetics outpatient clinics. *Am Heart J*. 2007;154:1130–1139.

13. Yuan W, Huang C, Wang J, Xie S, Nie R, Liu Y, Liu P, Zhou S, Chen S, Huang W. R25G mutation in exon 1 of LMNA gene is associated with dilated cardiomyopathy and limb-girdle muscular dystrophy 1B. *Chin Med J (Engl)*. 2009;122:2840–2845.

14. Parnham S, Selvanayagam JB, Haan E, Heddle W, De Pasquale CG. Lamin A/C mutation: An easily missed opportunity. *Int J Cardiol*. 2015;181:48–49.

15. Arbustini E, Pilotto A, Grasso M, Marziliano N, Serio A, Gambarin F, Pasotti M, Serafini E, Cassini P, Digiorgio B. Novel human pathological mutations. Gene symbol: LMNA. Disease: Cardiomyopathy, dilated with conduction defects. *Hum Genet*. 2009;125:350.

16. Arola AM, Li H, Murphy R, McKenna WJ, Bowles NE, Towbin JA. Clinical significance of lamin A/C mutations in dilated cardiomyopathy. *J Am Coll Cardiol*. 2004;43:A231 (1164-131).

17. Rudenskaya GE, Polyakov A V., Tverskaya SM, Zaklyazminskaya E V., Chukhrova AL, Groznova OE, Ginter EK. Laminopathies in Russian families. *Clin Genet*. 2008;74:127–133.

18. Song K, Dubé MP, Lim J, Hwang I, Lee I, Kim J-J. Lamin A/C mutations associated with familial and sporadic cases of dilated cardiomyopathy in Koreans. *Exp Mol Med*. 2007;39:114–120.

19. Verga L, Concardi M, Pilotto A, Bellini O, Pasotti M, Repetto A, Tavazzi L, Arbustini E. Loss of lamin A/C expression revealed by immuno-electron microscopy in dilated cardiomyopathy with atrioventricular block caused by LMNA gene defects. *Virchows Arch*. 2003;443:664–671.

20. McPherson E, Turner L, Zador I, Reynolds K, Macgregor D, Giampietro PF. Ovarian failure and dilated cardiomyopathy due to a novel lamin mutation. *Am J Med Genet A*. 2009;149A:567–572.

21. Fatkin D, MacRae C, Sasaki T, Wolff MR, Porcu M, Frenneaux M, Atherton J, Vidaillet HJ, Spudich S, De Girolami U, Seidman JG, Seidman C, Muntoni F, Müehle G, Johnson W, McDonough B, Hospital B, Clinic M, Medical H. Missense mutations in the rod domain of the lamin A/C gene as causes of dilated cardiomyopathy and conduction-system disease. *N Engl J Med*. 1999;341:1715–1724.

22. Garg A, Speckman RA, Bowcock AM. Multisystem dystrophy syndrome due to novel missense mutations in the amino-terminal head and alpha-helical rod domains of the lamin A/C gene. *Am J Med*. 2002;112:549–555.

23. Arbustini EA, Pasotti M, Pilotto A, Grasso M, Porcu E, Tocco G, Marziliano N. Gene symbol: LMNA. *Hum Genet*. 2007;120:907–908.

24. Kumar S, Androulakis AFA, Sellal J-M, Maury P, Gandjbakhch E, Waintraub X, Rollin A, Richard P, Charron P, Baldinger SH, Macintyre CJ, Koplan BA, John RM, Michaud GF, Zeppenfeld K, Sacher F, Lakdawala NK, Stevenson WG, Tedrow UB. Multicenter experience with catheter ablation for ventricular tachycardia in Lamin A/C cardiomyopathy. *Circ Arrhythmia Electrophysiol*. 2016;9:e004357.

25. Kourgiannidis G, Anastasakis A, Lampropoulos K, Iliopoulos T. A patient with ventricular tachycardia due to a novel mutation of the lamin A/C gene: Case presentation and mini review. *Hell J Cardiol*. 2013;54:326–330.

26. Wang H, Wang J, Zheng W, Wang X, Wang S, Song L, Zou Y, Yao Y, Hui R. Mutation Glu82Lys in lamin A/C gene is associated with cardiomyopathy and conduction defect. *Biochem Biophys Res Commun*. 2006;344:17–24.

27. Taylor MRG, Fain PR, Sinagra G, Robinson ML, Robertson AD, Carniel E, Di Lenarda A, Bohlmeyer TJ, Ferguson DA, Brodsky GL, Boucek MM, Lascor J, Moss AC, Li WLP, Stetler GL, Muntoni F, Bristow MR, Mestroni L, Dao D, Graw SL, Ku L, Lowes BD, Zhu X, Gowan K, Old WM, Driussi M, Scherl G. Natural history of dilated cardiomyopathy due to lamin A/C gene mutations. *J Am Coll Cardiol*. 2003;41:771–780.

28. Fokkema IFAC, den Dunnen JT, Taschner PEM. LOVD: easy creation of a locus-specific sequence variation database using an“LSDB-in-a-box” approach. *Hum Mutat*. 2005;26:63–68.

29. Millat G, Chanavat V, Julia S, Crehalet H, Bouvagnet P, Rousson R. Validation of high-resolution DNA melting analysis for mutation scanning of the LMNA gene. *Clin Biochem*. 2009;42:892–898.

30. Chami N, Tadros R, Lemarbre F, Lo KS, Beaudoin M, Robb L, Labuda D, Tardif J-C, Racine N, Talajic M, Lettre G. Nonsense mutations in BAG3 are associated with early-onset dilated cardiomyopathy in French Canadians. *Can J Cardiol*. 2014;30:1655–1661.

31. Arbustini E, Pilotto A, Repetto A, Grasso M, Negri A, Diegoli M, Campana C, Scelsi L, Baldini E, Gavazzi A, Tavazzi L. Autosomal dominant dilated cardiomyopathy with atrioventricular block: A lamin A/C defect-related disease. *J Am Coll Cardiol*. 2002;39:981–990.

32. Parks SB, Kushner JD, Nauman D, Ludwigsen S, Peterson A, Li D, Litt M, Porter CB, Rahko PS. Lamin A/C mutation analysis in a cohort of 324 unrelated patients with idiopathic or familial dilated cardiomyopathy. *Am Heart J*. 2009;156:161–169.

33. Botto N, Fontana M, Vittorini S, Colombo M, Manfredi S, Andreassi M. Expanding the cardiac phenotypes of laminopathies: novel LMNA mutations in two patients with left ventricular noncompaction before ventricular systolic dysfunction. *Abstr Eur Soc Cardiol Congr*. 2011;P1692.

34. Pan H, Richards AA, Zhu X, Joglar JA, Yin HL, Garg V. A novel mutation in LAMIN A/C is associated with isolated early-onset atrial fibrillation and progressive atrioventricular block followed by cardiomyopathy and sudden cardiac death. *Hear Rhythm*. 2009;6:707–710.

35. Keller H, Finsterer J, Steger C, Wexberg P, Gatterer E, Khazen C, Stix G, Gerull B, Höftberger R, Weidinger F. Novel c.367_369del LMNA mutation manifesting as severe arrhythmias, dilated cardiomyopathy, and myopathy. *Hear Lung J Acute Crit Care*. 2012;41:382–386.

36. Brauch KM, Chen LY, Olson TM. Comprehensive mutation scanning of LMNA in 268 patients with lone atrial fibrillation. *Am J Cardiol*. 2010;103:1426–1428.

37. Karkkainen S, Reissell E, Helio T, Kaartinen M, Tuomainen P, Toivonen L, Kuusisto J, Kupari M, Nieminen MS, Laakso M, Peuhkurinen K. Novel mutations in the lamin A/C gene in heart transplant recipients with end stage dilated cardiomyopathy. *Heart*. 2005;92:524–526.

38. Forleo C, Carmosino M, Resta N, Rampazzo A, Valecce R, Sorrentino S, Iacoviello M, Pisani F, Procino G, Gerbino A, Scardapane A, Simone C, Calore M, Torretta S, Svelto M, Favale S. Clinical and functional characterization of a novel mutation in lamin a/C gene in a multigenerational family with arrhythmogenic cardiac laminopathy. *PLoS One*. 2015;10:1–18.

39. Perrot A, Sigusch HH, Nägele H, Genschel J, Lehmkuhl H, Hetzer R, Geier C, Leon Perez V, Reinhard D, Dietz R, Josef Osterziel K, Schmidt HH-J. Genetic and phenotypic analysis of dilated cardiomyopathy with conduction system disease: demand for strategies in the management of presymptomatic lamin A/C mutant carriers. *Eur J Heart Fail*. 2006;8:484–493.

40. Kärkkäinen S, Heliö T, Miettinen R, Tuomainen P, Peltola P, Rummukainen J, Ylitalo K, Kaartinen M, Kuusisto J, Toivonen L, Nieminen MS, Laakso M, Peuhkurinen K. A novel mutation, Ser143Pro, in the lamin A/C gene is common in finnish patients with familial dilated cardiomyopathy. *Eur Heart J*. 2004;25:885–893.

41. Sims-Williams HP, Nye HJ, Walker PR. Dilated cardiomyopathy and skeletal myopathy: presenting features of a laminopathy. *BMJ Case Rep*. 2013.

42. Nishi T, Takaoka H, Funabashi N, Nishimura M, Ohara O, Makiyama T, Ueda M, Kajiyama T, Kobayashi Y. Familial lamin A/C mutation cardiomyopathy with arrhythmia substrate detected by cardiac magnetic resonance imaging and electroanatomical mapping. *Int J Cardiol*. 2016;209:248–252.

43. Hirtle-Lewis M, Desbiens K, Ruel I, Rudzicz N, Genest J, Engert JC, Giannetti N. The genetics of dilated cardiomyopathy: A prioritized candidate gene study of LMNA, TNNT2, TCAP, and PLN. *Clin Cardiol*. 2013;36:628–633.

44. Brodsky GL, Muntoni F, Miocic S, Sinagra G, Sewry C, Mestroni L. Lamin A/C gene mutation associated with dilated cardiomyopathy with variable skeletal muscle involvement. *Circulation*. 2000;101:473–476.

45. Chen C-H, Tang S-C, Su Y-N, Yang C-C, Jeng J-S. Cardioembolic stroke related to limb-girdle muscular dystrophy 1B. *BMC Res Notes*. 2013;6:32.

46. Gupta P, Bilinska ZT, Sylvius N, Boudreau E, Veinot JP, Labib S, Bolongo PM, Hamza A, Jackson T, Ploski R, Walski M, Grzybowski J, Walczak E, Religa G, Fidzianska A, Tesson F. Genetic and ultrastructural studies in dilated cardiomyopathy patients: A large deletion in the lamin A/C gene is associated with cardiomyocyte nuclear envelope disruption. *Basic Res Cardiol*. 2010;105:365–377.

47. Botto N, Vittorini S, Colombo MG, Biagini A, Paradossi U, Aquaro G, Andreassi MG. A novel LMNA mutation (R189W) in familial dilated cardiomyopathy: evidence for a “hot spot” region at exon 3: a case report. *Cardiovasc Ultrasound*. 2010;8:9.

48. Marsman RF, Bardai A, Postma A V., Res JCJ, Koopmann TT, Beekman L, Van Der Wal AC, Pinto YM, Deprez RHL, Wilde AAM, Jordaens LJ, Bezzina CR. A complex double deletion in LMNA underlies progressive cardiac conduction disease, atrial arrhythmias, and sudden death. *Circ Cardiovasc Genet*. 2011;4:280–287.

49. Subramanyam L, Simha V, Garg A. Overlapping syndrome with familial partial lipodystrophy, Dunnigan variety and cardiomyopathy due to amino-terminal heterozygous missense lamin A/C mutations. *Clin Genet*. 2012;100:130–134.

50. Sylvius N, Bilinska ZT, Veinot JP, Fidzianska A, Bolongo PM, Poon S, McKeown P, Davies RA, Chan K-L, Tang ASL, Dyack S, Grzybowski J, Ruzyllo W, McBride H, Tesson F. In vivo and in vitro examination of the functional significances of novel lamin gene mutations in heart failure patients. *J Med Genet*. 2005;42:639–647.

51. Jakobs PM, Hanson EL, Crispell K a, Toy W, Keegan H, Schilling K, Icenogle TB, Litt M, Hershberger RE. Novel lamin A/C mutations in two families with dilated cardiomyopathy and conduction system disease. *J Card Fail*. 2001;7:249–256.

52. Perrot A, Hussein S, Ruppert V, Schmidt HHJ, Wehnert MS, Duong NT, Posch MG, Panek A, Dietz R, Kindermann I, Böhm M, Michalewska-Wludarczyk A, Richter A, Maisch B, Pankuweit S, Özcelik C. Identification of mutational hot spots in LMNA encoding lamin A/C in patients with familial dilated cardiomyopathy. *Basic Res Cardiol*. 2009;104:90–99.

53. Otomo J, Kure S, Shiba T, Karibe A, Shinozaki T, Yagi T, Naganuma H, Tezuka F, Miura M, Ito M, Watanabe J, Matsubara Y, Shirato K. Electrophysiological and histopathological characteristics of progressive atrioventricular block accompanied by familial dilated cardiomyopathy caused by a novel mutation of lamin A/C gene. *J Cardiovasc Electrophysiol*. 2005;16:137–145.

54. Hershberger RE, Hanson EL, Jakobs PM, Keegan H, Coates K, Bousman S, Litt M. A novel lamin A/C mutation in a family with dilated cardiomyopathy, prominent conduction system disease, and need for permanent pacemaker implantation. *Am Heart J*. 2002;144:1081–1086.

55. Rasmussen T, Al-Saaidi R, Birkler RI, Palmfeldt J, Beqqali A, Pinto Y, Baandrup U, Moelgaard H, Hey T, Eiskjaer H, Bross P, Mogensen J. Lamin A/C missense mutations causing cardiomyopathy are associated with highly variable outcomes despite uniform disease mechanisms. *Eur Heart J*. 2017;38:P1607.

56. Bonne G, Mercuri E, Muchir A, Urtizberea A, Bécane HM, Recan D, Merlini L, Wehnert M, Boor R, Reuner U, Vorgerd M, Wicklein EM, Eymard B, Duboc D, Penisson-Besnier I, Cuisset JM, Ferrer X, Desguerre I, Lacombe D, Bushby K, Pollitt C, Toniolo D, Fardeau M, Schwartz K, Muntoni F. Clinical and molecular genetic spectrum of autosomal dominant Emery-Dreifuss muscular dystrophy due to mutations of the lamin A/C gene. *Ann Neurol*. 2000;48:170–180.

57. Ito K, McDonough B, Gorham JM, DeParma SR, Adler EE, Mohiuddin SM, Fatkin D, Seidman JG, Seidman CE. Abstract 17982: A lamin A/C synonymous mutation creates a novel splice site and causes progressive atrioventricular conduction defect. *Circulation*. 2014;130:A17982 LP-A17982.

58. Saga A, Karibe A, Otomo J, Iwabuchi K, Takahashi T, Kanno H, Kikuchi J, Keitoku M, Shinozaki T, Shimokawa H. Lamin A/C gene mutations in familial cardiomyopathy with advanced atrioventricular block and arrhythmia. *Tohoku J Exp Med*. 2009;218:309–316.

59. Arbustini Eloisa AE, Pilotto A, Pasotti M, Grasso M, Diegoli M, Campana C, Gavazzi A, Alessandra R, Tavazzi L. Gene symbol: LMNA. Disease: Cardiomyopathy, dilated, with conduction defect 1. *Hum Genet*. 2005;117:298.

60. Felice KJ, Schwartz RC, Brown CA, Leicher CR, Grunnet ML. Autosomal dominant Emery-Dreifuss dystrophy due to mutations in rod domain of the lamin A/C gene. *Neurology*. 2000;55:275–280.

61. Carboni N, Sardu C, Cocco E, Marrosu G, Manzi RC, Nissardi V, Isola F, Mateddu A, Solla E, Maioli MA, Oppo V, Piras R, Coghe G, Lai C, Marrosu MG. Cardiac involvement in patients with lamin A/C gene mutations: A cohort observation. *Muscle and Nerve*. 2012;46:187–192.

62. Beckmann BM, Holinski-Feder E, Walter MC, Haserück N, Reithmann C, Hinterseer M, Wilde AA, Kääb S. Laminopathy presenting as familial atrial fibrillation. *Int J Cardiol*. 2010;145:394–396.

63. Karrouz W, Lemaire C, Douillard C, Launay D, Vantyghem MC, Lacroix D, Launay D. P251 Laminopathie atypique revele par un diabete non insulino dependant et des troubles de conduction. In: Diabetes & Metabolism. 2009. p. A86.

64. Finsterer J, Stöllberger C, Keller H, Gencik M. Familial accumulation of sudden cardiac deaths and the LMNA variant c.868G > A (p.Glu290Lys). *Int J Cardiol*. 2016;215:84–86.

65. Pérez-Serra A, Toro R, Campuzano O, Sarquella-Brugada G, Berne P, Iglesias A, Mangas A, Brugada J, Brugada R. A novel mutation in lamin A/C causing familial dilated cardiomyopathy associated with sudden cardiac death. *J Card Fail*. 2015;21:217–225.

66. Ben Yaou R Ben, Toutain A, Arimura T, Demay L, Massart C, Peccate C, Muchir A, Llense S, Deburgrave N, Leturcq F, Litim KE, Rahmoun-Chiali N, Richard P, Babuty D, Récan-Budiartha D, Bonne G, Ben Yaou R, Toutain A, Arimura T, Demay L, Massart C, Peccate C, Muchir A, Llense S, Deburgrave N, Leturcq F, Litim KE, Rahmoun-Chiali N, Richard P, Babuty D, Récan-Budiartha D, Bonne G. Multitissular involvement in a family with LMNA and EMD mutations: Role of digenic mechanism? *Neurology*. 2007;68:1883–1894.

67. Antoniades L, Eftychiou C, Kyriakides T, Christodoulou K, Katritsis DG. Malignant mutation in the lamin A/C gene causing progressive conduction system disease and early sudden death in a family with mild form of limb-girdle muscular dystrophy. *J Interv Card Electrophysiol*. 2007;19:1–7.

68. Ben Yaou R, Bécane H-M, Demay L, Laforet P, Hannequin D, Bohu P-A, Drouin-Garraud V, Ferrer X, Mussini J-M, Ollagnon E, Petiot P, Penisson-Besnier I, Streichenberger N, Toutain A, Richard P, Eymard B, Bonne G. Autosomal dominant limb-girdle muscular dystrophy associated with conduction defects (LGMD1B): a description of 8 new families with the LMNA gene mutations. *Rev Neurol (Paris)*. 2005;161:42–54.

69. Van Spaendonck-Zwarts KY, Van Rijsingen IAW, Van Den Berg MP, Lekanne Deprez RH, Post JG, Van Mil AM, Asselbergs FW, Christiaans I, Van Langen IM, Wilde AAM, De Boer RA, Jongbloed JDH, Pinto YM, Van Tintelen JP. Genetic analysis in 418 index patients with idiopathic dilated cardiomyopathy: Overview of 10 years’ experience. *Eur J Heart Fail*. 2013;15:628–636.

70. Carboni N, Floris M, Mateddu A, Porcu M, Marrosu G, Solla E, Cocco E, Mura M, Marini S, Maioli MA, Piras R, Aste R, Marrosu MG. Aberrant splicing in the LMNA gene caused by a novel mutation on the polypyrimidine tract of intron 5. *Muscle and Nerve*. 2011;43:688–693.

71. Banerjee A, Ghoshal PK, Sengupta K. Novel linkage of LMNA single nucleotide polymorphism with dilated cardiomyopathy in an Indian case study. *Int J Cardiol Hear Vasc*. 2015;7:99–105.

72. Hasselberg NE, Edvardsen T, Petri H, Berge KE, Leren TP, Bundgaard H, Haugaa KH. Risk prediction of ventricular arrhythmias and myocardial function in Lamin A/C mutation positive subjects. *Europace*. 2014;16:563–571.

73. Benedetti S, Menditto I, Degano M, Rodolico C, Merlini L, D’Amico A, Palmucci L, Berardinelli A, Pegoraro E, Trevisan CP, Morandi L, Moroni I, Galluzzi G, Bertini E, Toscano A, Olivè M, Bonne G, Mari F, Caldara R, Fazio R, Mammì I, Carrera P, Toniolo D, Comi G, Quattrini A, Ferrari M, Previtali SC. Phenotypic clustering of lamin A/C mutations in neuromuscular patients. *Neurology*. 2007;69:1285–1292.

74. Hoorntje ET, Bollen IA, Barge-Schaapveld DQ, van Tienen FH, Te Meerman GJ, Jansweijer JA, van Essen AJ, Volders PG, Constantinescu AA, van den Akker PC, van Spaendonck-Zwarts KY, Oldenburg RA, Marcelis CL, van der Smagt JJ, Hennekam EA, Vink A, Bootsma M, Aten E, Wilde AA, van den Wijngaard A, Broers JL, Jongbloed JD, van der Velden J, van den Berg MP, van Tintelen JP. Lamin A/C-related cardiac disease: late onset with a variable and mild phenotype in a large cohort of patients with the lamin A/C p.(Arg331Gln) founder mutation. *Circ Cardiovasc Genet*. 2017;10.

75. Stallmeyer B, Koopmann M, Schulze-Bahr E. Identification of novel mutations in LMNA associated with familial forms of dilated cardiomyopathy. *Genet Test Mol Biomarkers*. 2012;16:543–549.

76. Ehlermann P, Georgiev K, Ivandic B, Zeller R, Pribe R, Zugck C, Remppis A, Gruenig E, Weichenhan D, Katus HA. Lamin A/C mutations in patients with dilated cardiomyopathy and conduction disease, ventricular dysrhythmia or family history of sudden death. *Eur Soc Cardiol Congr*. 2009;Abstract.

77. Vytopil M, Benedetti S, Ricci E, Galluzzi G, Dello Russo A, Merlini L, Boriani G, Gallina M, Morandi L, Politano L, Moggio M, Chiveri L, Hausmanova-Petrusewicz I, Ricotti R, Vohanka S, Toman J, Toniolo D. Mutation analysis of the lamin A/C gene (LMNA) among patients with different cardiomuscular phenotypes. *J Med Genet*. 2003;40:e132.

78. Bollati M, Barbiroli A, Favalli V, Arbustini E, Charron P, Bolognesi M. Structures of the lamin A/C R335W and E347K mutants: Implications for dilated cardiolaminopathies. *Biochem Biophys Res Commun*. 2012;418:217–221.

79. Meinke P, Nguyen TD, Wehnert MS. The LINC complex and human disease. *Biochem Soc Trans*. 2011;39:1693–1697.

80. Hermida-Prieto M, Monserrat L, Castro-Beiras A, Laredo R, Soler R, Peteiro J, Rodríguez E, Bouzas B, Alvarez N, Muñiz J, Crespo-Leiro M. Familial dilated cardiomyopathy and isolated left ventricular noncompaction associated with lamin A/C gene mutations. *Am J Cardiol*. 2004;94:50–54.

81. Fujimori Y, Okimatsu H, Kashiwagi T, Sanda N, Okumura K, Takagi A, Nagata K, Murate T, Uchida A, Node K, Saito H, Kojima T. Molecular defects associated with antithrombin deficiency and dilated cardiomyopathy in a Japanese patient. *Intern Med*. 2008;47:925–31.

82. Dittmer T, Sahni N, Kubben N, Hill DE, Vidal M, Burgess RC, Roukos V, Misteli T. Systematic identification of pathological lamin A interactors. *Mol Biol Cell*. 2014;25:1493–1510.

83. De Backer J, Van Beeumen K, Loeys B, Duytschaever M. Expanding the phenotype of sudden cardiac death-An unusual presentation of a family with a Lamin A/C mutation. *Int J Cardiol*. 2010;138:97–99.

84. Komaki H, Hayashi YK, Tsuburaya R, Sugie K, Kato M, Nagai T, Imataka G, Suzuki S, Saitoh S, Asahina N, Honke K, Higuchi Y, Sakuma H, Saito Y, Nakagawa E, Sugai K, Sasaki M, Nonaka I, Nishino I. Inflammatory changes in infantile-onset LMNA-associated myopathy. *Neuromuscul Disord*. 2011;21:563–568.

85. Anselme F, Moubarak G, Savouré A, Godin B, Borz B, Drouin-Garraud V, Gay A. Implantable cardioverter-defibrillators in lamin A/C mutation carriers with cardiac conduction disorders. *Hear Rhythm*. 2013;10:1492–1498.

86. Charniot JC, Pascal C, Bouchier C, Sébillon P, Salama J, Duboscq-Bidot L, Peuchmaurd M, Desnos M, Artigou JY, Komajda M. Functional consequences of an LMNA mutation associated with a new cardiac and non-cardiac phenotype. *Hum Mutat*. 2003;21:473–481.

87. Quenin P, Kyndt F, Mabo P, Mansourati J, Babuty D, Thollet A, Guyomarch B, Redon R, Barc J, Schott J-J, Sacher F, Probst V, Gourraud JB. Clinical yield of familial screening after sudden death in young subjects: the French experience. *Circ Arrhythm Electrophysiol*. 2017;10:e005236.

88. Møller DV, Pham TT, Gustafsson F, Hedley P, Ersbøll MK, Bundgaard H, Andersen CB, Torp-Pedersen C, Køber L, Christiansen M. The role of Lamin A/C mutations in Danish patients with idiopathic dilated cardiomyopathy. *Eur J Heart Fail*. 2009;11:1031–1035.

89. Perrot A, Neubert M, Dietz R, Posch M, Oezcelik C. A novel lamin A/C mutation in hypertrophic cardiomyopathy illustrates the diversity of cardiomyopathy phenotypes in the laminopathies. *Front Cardiovasc Biol Berlin, First Congr ESC Counc Basic Cardiovasc Sci*. 2010;Abstract.

90. Scharner J, Lu HC, Fraternali F, Ellis JA, Zammit PS. Mapping disease-related missense mutations in the immunoglobulin-like fold domain of lamin A/C reveals novel genotype-phenotype associations for laminopathies. *Proteins Struct Funct Bioinforma*. 2014;82:904–915.

91. Genschel J, Baier P, Kuepferling S, Proepsting MJ, Buettner C, Ewert R, Hetzer R, Lochs H, Schmidt HH. A new frameshift mutation at codon 466 (1397delA) within the LMNA gene. *Hum Mutat*. 2000;151:1–3.

92. Duparc A, Cintas P, Somody E, Bieth E, Richard P, Maury P, Delay M. A cardio-neurological form of laminopathy: dilated cardiomyopathy with permanent partial atrial standstill and axonal neuropathy. *Pacing Clin Electrophysiol*. 2009;32:410–415.

93. Chen W, Huo J, Ma A, Bai L, Liu P. A novel mutation of the LMNA gene in a family with dilated cardiomyopathy, conduction system disease, and sudden cardiac death of young females. *Mol Cell Biochem*. 2013;382:307–311.

94. Saj M, Bilinska ZT, Tarnowska A, Sioma A, Bolongo P, Sobieszczanska-Malek M, Michalak E, Golen D, Mazurkiewicz L, Malek L, Walczak E, Fidzianska A, Grzybowski J, Przybylski A, Zielinski T, Korewicki J, Tesson F, Ploski R. LMNA mutations in Polish patients with dilated cardiomyopathy: prevalence, clinical characteristics, and in vitro studies. *BMC Med Genet*. 2013;14:55.

95. Te Rijdt WP, van der Klooster ZJ, Hoorntje ET, Jongbloed JDH, van der Zwaag PA, Asselbergs FW, Dooijes D, de Boer RA, van Tintelen JP, van den Berg MP, Vink A, Suurmeijer AJH. Phospholamban immunostaining is a highly sensitive and specific method for diagnosing phospholamban p.Arg14del cardiomyopathy. *Cardiovasc Pathol*. 2017;30:23–26.

96. Renou L, Stora S, Yaou R Ben, Volk M, Sinkovec M, Demay L, Richard P, Peterlin B, Bonne G. Heart-hand syndrome of Slovenian type: a new kind of laminopathy. *J Med Genet*. 2008;45:666–671.

97. Chrestian N, Valdmanis PN, Echahidi N, Brunet D, Bouchard J-P, Gould P, Rouleau GA, Champagne J, Dupré N. A novel mutation in a large French-Canadian family with LGMD1B. *Can J Neurol Sci*. 2008;35:331–334.

98. Olaopa MA, Spoonamore KG, Bhakta D, Chen Z, Celestino-Soper PBS, Chen P-S, Ai T, Vatta M. Lamin-A/C variants found in patients with cardiac conduction disease reduce sodium currents. *Cardiogenetics*. 2018;8:316–324.

99. Benedetti S, Menditto I, Rodolico C, Carrera P, Toniolo D, Ferrari M. Distribution and characterization of lamin A/C mutations: a role for silent variations? *Am Soc Hum Genet Annu Meet*. 2004;Abstrct.

100. Pugh TJ, Kelly MA, Gowrisankar S, Hynes E, Seidman MA, Baxter SM, Bowser M, Harrison B, Aaron D, Mahanta LM, Lakdawala NK, McDermott G, White ET, Rehm HL, Lebo M, Funke BH. The landscape of genetic variation in dilated cardiomyopathy as surveyed by clinical DNA sequencing. *Genet Med*. 2014;16:601.

101. Genschel J, Bochow B, Kuepferling S, Ewert R, Hetzer R, Schmidt HH, Mitte CC, Berlin DH, Mitte CC. A R644C mutation within lamin A extends the mutations causing dilated cardiomyopathy. *Hum Mutat*. 2000;201:65–68.

102. Moller D V, Behr ER, Hedley P, Syrris P, Elliott PM, McKenna W J, Christiansen M. Mutations in the Lamin A/C gene play a major role in sudden arrhythmic death syndrome. *Circulation*. 2010;122:A16108.

103. Arbustini E, Narula N, Tavazzi L, Serio A, Grasso M, Favalli V, Bellazzi R, Tajik JA, Bonow RDO, Fuster V, Narula J, Bonow RDO, Fuster V, Narula J. The MOGE(S) classification of cardiomyopathy for clinicians. *J Am Coll Cardiol*. 2014;64:304–318.
